# Improving antibiotic stewardship skills through interdisciplinary online education: a multicenter teaching intervention for physicians and pharmacists

**DOI:** 10.1186/s12909-025-08348-4

**Published:** 2025-12-17

**Authors:** Till Koch, Johannes Jochum, Annika van der Linde, Friederike Jahn, Anika Neubert, Evelyn Kramme, Anette Friedrichs

**Affiliations:** 1 Antibiotic Stewardship Team, Pharmacy of the University Medical Center Hamburg- Eppendorf, Hamburg, Germany; 2 3rd Medical Clinic for Pneumology, Infectious Diseases and Oncology, Klinikum Itzehoe, Itzehoe, 25524 Germany; 3 German Center for Infection Research (DZIF), Partner Site Hamburg-Lübeck-Borstel-Riems, Hamburg, Germany; 4 1 st Department of Medicine, University Medical Center Hamburg-Eppendorf, Hamburg, Germany; 5 Center for Tropical Medicine, Bernhard Nocht Institute for Tropical Medicine, Hamburg, Germany; 6 Antibiotic Stewardship Team, Pharmacy of the University Medical Center Schleswig- Holstein, Campus Kiel, Kiel, Germany; 7 Antibiotic Stewardship Team, Pharmacy of the University Medical Center Schleswig- Holstein, Campus Lübeck, Lübeck, Germany; 8 Antibiotic Stewardship Team, Commercial Management, University Medical Center Schleswig-Holstein, Campus Lübeck, Lübeck, Germany; 9 Department of Infectious Diseases, University Medical Center Schleswig-Holstein, Campus Lübeck, Lübeck, Germany; 10 Antibiotic Stewardship Team, Commercial Management, University Medical Center Schleswig-Holstein, Campus Kiel, Kiel, Germany; 11 1st Department of Medicine, University Medical Center Schleswig-Holstein, Campus Kiel, Kiel, Germany

**Keywords:** Antibiotics, Resistance, Education, Learning, Interdisciplinarity

## Abstract

**Background:**

Effective antibiotic prescribing is a core competency for physicians and pharmacists in combating antimicrobial resistance. Despite this, many healthcare professionals report limited confidence in applying antibiotic stewardship principles in clinical practice. This study evaluates the impact of a structured, interdisciplinary online course designed to improve knowledge and self-efficacy in rational antibiotic use.

**Methods:**

We developed and implemented a problem-based, eight-module online course delivered across four hospitals in 2023 and 2024. The course targeted physicians and pharmacists and focused on practical aspects of antimicrobial prescribing. In a quasi-experimental pre-post design, participants completed self-assessments regarding their confidence and knowledge in antibiotic stewardship using a validated 25-item questionnaire.

**Results:**

Across both years, an average of 95 participants per session completed the course. Post-course evaluations showed a consistent increase in self-reported confidence levels in managing antibiotic therapy. On a seven-stepped scale (1 = very well prepared; 7 = not prepared at all), confidence increased from 3.53 (95% CI 3.3–3.8) to 2.16 (95% CI 2.0–2.3). Participants highlighted the relevance of interprofessional exchange and case-based discussions as key benefits.

**Conclusion:**

Structured online education with interdisciplinary participation and active learning strategies can enhance healthcare professionals’ confidence and competence in antibiotic stewardship. Regular evaluation of both content delivery and learner outcomes is essential to optimize such training formats and support responsible antimicrobial prescribing in clinical settings.

**Supplementary Information:**

The online version contains supplementary material available at 10.1186/s12909-025-08348-4.

## Background

Antimicrobial resistance (AMR) is a growing global public health threat and bacterial AMR were attributable for more than 1,2 million annual deaths globally in 2019 [1]. Inadequate use of antibiotics by healthcare workers (HCW) is one of the leading factors in the development of bacterial AMR [2]. The growing prevalence of multidrug-resistant organisms has prompted the need for effective antimicrobial stewardship (AMS) programs to optimize the use of antibiotics, improve patient outcomes, and reduce resistance. Various AMS interventions have been implemented in clinical practice [3].

Education and training are widely recognized as critical components of AMS interventions. In particular, behavioural change through active, learner-centred methods has been shown to be a key factor in promoting sustainable prescribing practices [4]. A solid understanding of anti-infective therapy is essential for rational antibiotic use; however, structured AMS education for clinicians, pharmacists, and medical students remains limited — despite increasing evidence of educational needs across these groups.

In Germany, AMS efforts are addressed both by physicians and pharmacists. While it is reserved for physicians to diagnose infectious diseases and prescribe antimicrobial agents, pharmacists have a pivotal role in antimicrobial use, as they are an important and readily accessible source of information for patients.

In 2018, the first multi-country and multi-professional study to assess the knowledge, attitude and behaviours of HCW on antibiotics, antibiotic use and antibiotic resistance was launched by the European Centre for Disease Prevention and Control. German participants in particular highlighted published guidelines as a primary information source, and the study recommended prioritizing educational efforts for HCWs who reported low confidence in antibiotic management [5]. Additional surveys have shown that non-commercial, digital learning formats — such as E-learning courses — are well received by German physicians [6]. Moreover, several studies have identified substantial gaps in prescribing confidence and antimicrobial knowledge among medical students [7].

Furthermore, evidence suggests that active learning formats such as problem-based or case-based learning can significantly improve the appropriateness of antibiotic prescribing compared to traditional lecture-based methods [8]. Lastly, E-learning modules on AMS have also been demonstrated to deliver outcomes comparable to those of in-person training sessions [9].

Against this backdrop, we developed, implemented, and evaluated an interdisciplinary, structured, multicenter, annual online course on antimicrobial stewardship.

Our course was grounded in Adult Learning Theory [10] and Social Cognitive Theory [11], using pre-question prompts, practice-centered content, and feedback (including live chat Q&A) to build self-efficacy in antimicrobial stewardship.

This study evaluated the impact of the online- course on self-efficacy in rational antibiotic use.

## Methods

### Course development and design

An interdisciplinary online teaching course on antimicrobial stewardship (AMS) was conceptualized and developed between January and August 2023 by an organizing committee (OC) comprising infectious disease specialists and hospital pharmacists from three institutions in Northern Germany: University Hospital Schleswig-Holstein (UKSH) Campus Kiel, UKSH Campus Lübeck and the University Medical Center Hamburg-Eppendorf (UKE). The OC consisted of the authors of this paper.

The course was based on previous in-person courses held in prior years but redesigned in response to participant feedback. Learners had previously reported challenges related to limited pharmacological background and insufficient practical guidance for clinical decision-making. To address this, the OC developed a revised course structure focusing on foundational AMS content, active learning methods, and consistent didactic delivery.

Key design principles included:

- A maximum session duration of 60 min (including discussion).
- Division of each session into a pharmacological section and a clinically/microbiologically focused section.
- Delivery by AMS team members from participating institutions.
- Harmonized presentation format and visual layout.
- Course participation easily accessible and free of charge, at after-work hours for most participants (weekdays, 6–7 PM).

**Fig. 1:**
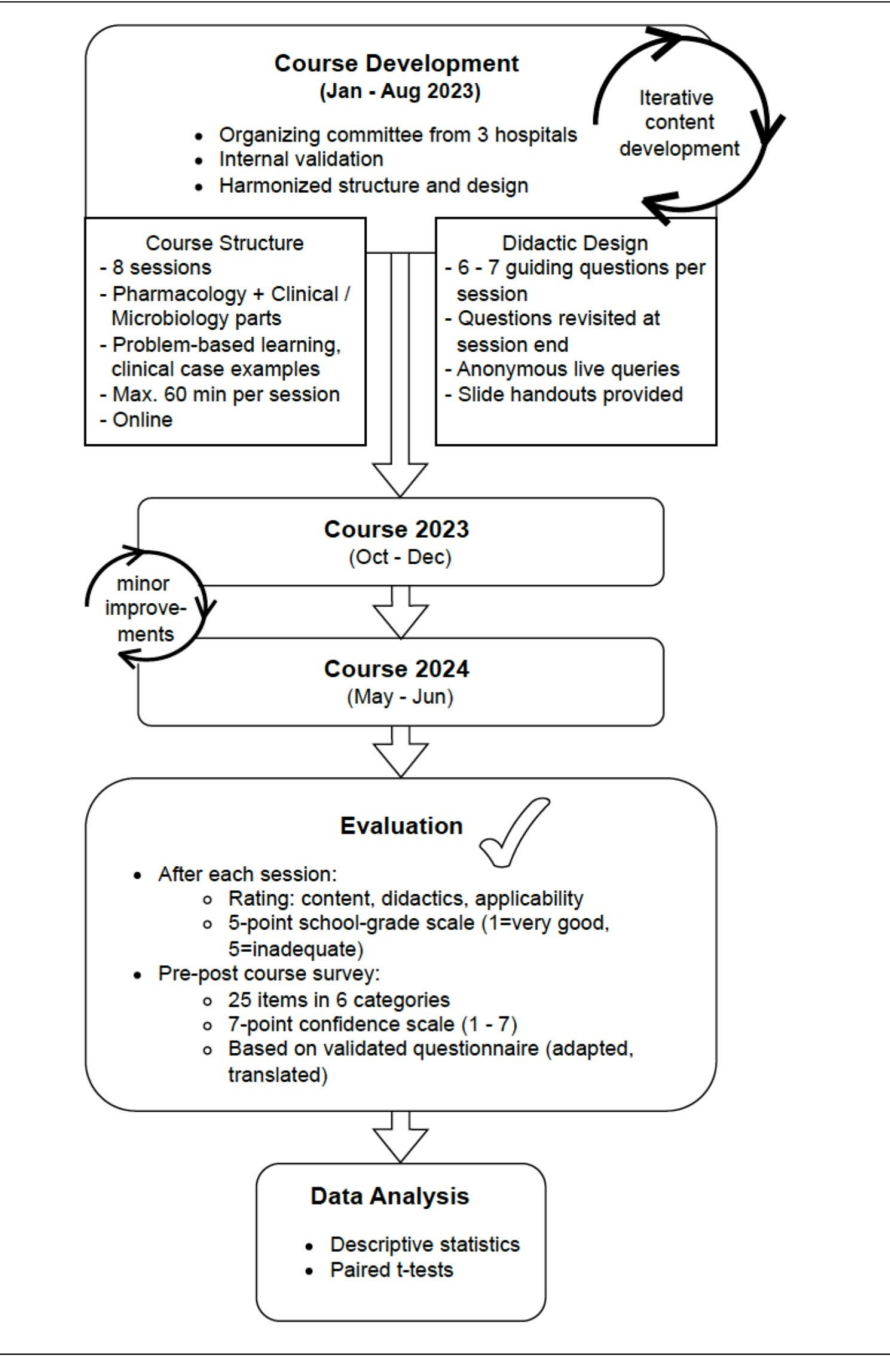
Structure and Evaluation Workflow of the Interdisciplinary AMS Online Course. This flowchart illustrates the development, structure, delivery, and evaluation of the interdisciplinary online teaching course on antimicrobial stewardship (AMS) conducted in 2023 and 2024 by three university hospitals in Northern Germany. The course was designed using an iterative course improvement. Key features included problem-based learning principles and a standardized format, combining pharmacological and clinical/microbiological components. Evaluation included session-based feedback and a pre–post survey measuring self-reported confidence in antibiotic management. Data analysis was conducted using descriptive statistics and paired *t*-tests

Course content was developed through an iterative peer-review process. A draft presentation was prepared by an OC member, reviewed and revised within the OC, and subsequently validated by the broader AMS teams of all three institutions. Emphasis was placed on aligning core messages, avoiding cognitive overload, and standardizing didactic techniques. An overview of the course’s structure and evaluation workflow is shown in Fig. 1.

### Theoretical framework

The instructional design was informed by Adult Learning Theory (problem- and practice-centered, immediately relevant content) and Social Cognitive Theory, targeting self-efficacy through (i) mastery experiences (worked explanations aligning with the pre-posed questions), (ii) vicarious learning (expert reasoning during the clinical segment), and (iii) verbal feedback (end-of-part answer reveal and live Q&A). The “questions-first, answers-later” structure also leverages the retrieval-practice/pre-question effect, which focuses attention and supports durable learning [12]. Our evaluation focused on Kirkpatrick Levels 1–2 (Reaction and Learning/Self-efficacy).

### Course delivery and structure

The course was delivered in eight sessions between October and December 2023, and repeated between May and June 2024. Content remained largely consistent across both iterations, with minor didactic refinements and session reordering due to speaker availability; a brief summary of the eight session’s content of the 2024 course can be found in the supplement.

Sessions employed problem-based learning (PBL) methodologies, including clinical case scenarios with interactive audience questions. Online query tools enabled real-time, anonymous participation; responses were not recorded to foster a safe learning environment.

**Fig. 2:**
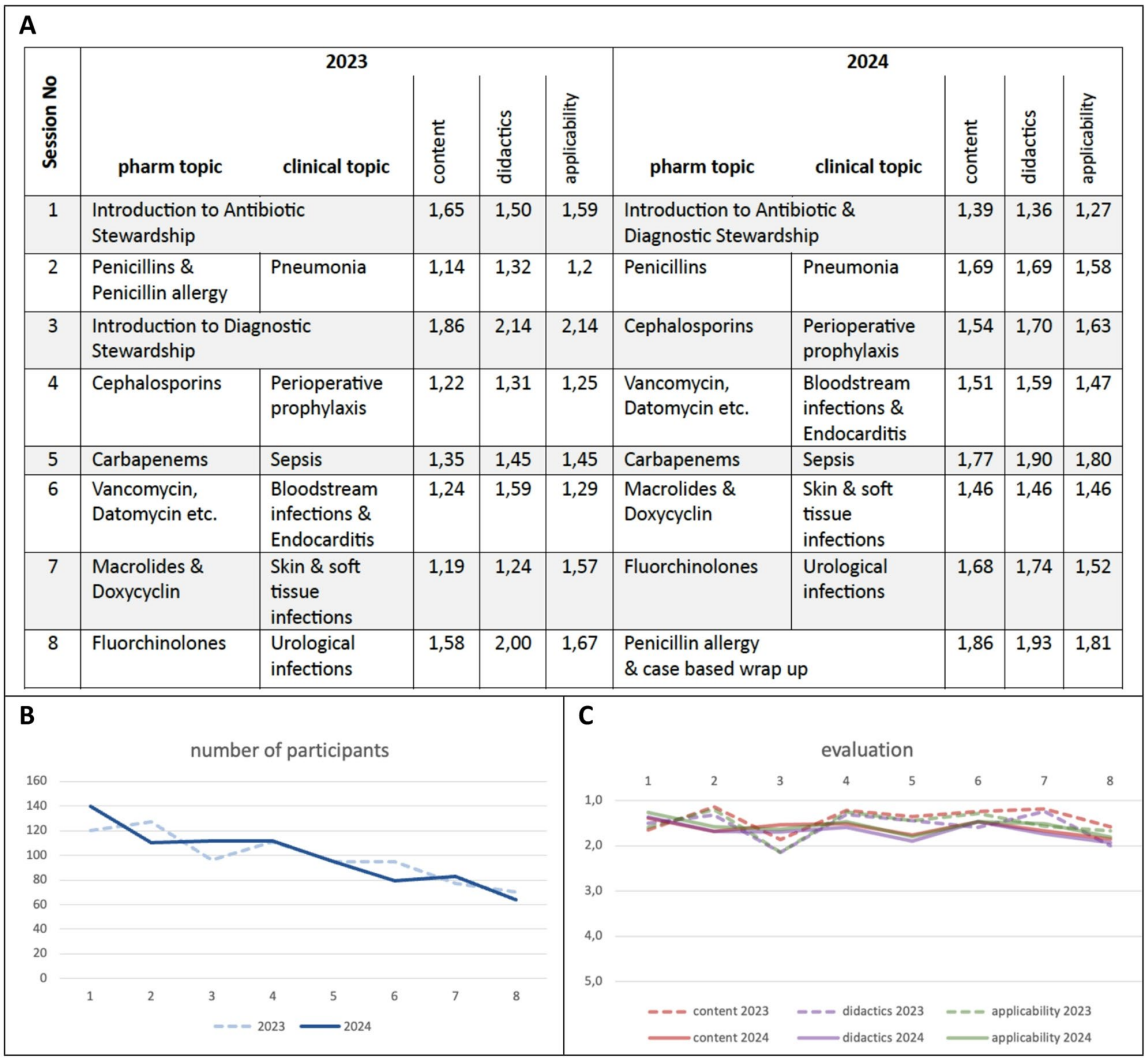
Course content, number of participants and evaluation. **A** Overview of the session topics in 2023 and 2024 including the numerical mean session rating scores for each session regarding content, didactics and applicability. **B** Number of participants (Y-axis) per session (X-axis, numbered 1 to 8) over time in 2023 (dashed line) and 2024 (continuous line). **C** Graphical presentation of the *mean session rating* scores of content (red), didactics (violet), and applicability (green) (Y-axis) per session (X-axis, numbered 1 to 8) in 2023 (dashed lines) and 2024 (continuous lines), based on a 5-point school grading scale where lower scores signify better performance (1 = very good, 5 = inadequate)

Each session was structured around key learning questions presented at the beginning and revisited at the end for summary and reinforcement. Sessions with both pharmacological and clinical parts included up to seven guiding questions (three to four per section, not answered immediately). A full list of session topics is provided in Fig. 2A; the learning questions are available in the Supplement. The subsequent teaching in that part provided the information needed to answer those questions; at the end of the part, the questions were revisited and model answers were presented.

The sessions were conducted live via online conferencing platforms (Webex in 2023; Zoom in 2024), which enabled the recording of participation numbers. No recording of the session was performed for privacy reasons as we did not wish to ask permission for recording from attendees. Each session in the course was held by two lecturers (one pharmacist and one medical doctor) and moderated by one facilitator. Learners’ questions were collected and addressed in a moderated discussion at the end of each session, allowing participants to receive immediate feedback. PDF slide handouts were provided after each session.

For each course, we invited physicians, pharmacists and last-year students from both pharmacy and medicine from all participating institutions via email.

As part of a quality control measure for the online course, a detailed evaluation of the course was performed. All data was collected anonymously. In this study, no personal or identifiable data were collected, and the analysis was limited to aggregated, non-traceable survey responses. Therefore, in accordance with our local Ethics Committee, ethical approval was not required under German law and common academic practice.

### Session evaluation

After each session of the eight-session course, participants were asked to rate three items, i.e. quality of content, didactic clarity, and practical applicability of this day’s session using a five-point scale based on the German school grading system, where lower scores signify better performance (1 = very good, 5 = inadequate). The *mean session rating scores* for content, didactics, and applicability were calculated by creating the mean of all participants’ ratings per item per session. Subsequently, to further aggregate the evaluation data, a *mean overall session score* was calculated by creating the mean of all eight session’s mean per item.

### Study design

A quasi-experimental study design without a control group was used to assess the educational intervention, as randomization was not feasible. Data collection was performed using pre- and post-course surveys developed to assess self-reported knowledge, attitudes, and intended behaviors related to antimicrobial stewardship. These surveys were administered immediately before and after the intervention.

### Pre–post course survey on learner confidence

To evaluate the overall impact of the course on learners’ confidence in antibiotic management, a pre–post survey was administered using REDCap. The survey was adapted from a previously validated questionnaire [13], assessing self-perceived preparedness across six domains:

A) Diagnosis of infection
B) Indications for withholding antibiotics
C) Initiation of antibiotic therapy
D) Reassessment of therapy
E) Communication skills
F) Antimicrobial resistance

The original survey included 27 items across seven domains; for this study, it was translated into German, adapted for the course context, and reduced to 25 items by excluding two items from the “Quality of care” domain, which were not applicable in our teaching context.

Responses were collected on a 7-point Likert scale where lower scores indicated greater perceived preparedness (1 = very well prepared, 4 = sufficiently prepared, 7 = not prepared at all). The pre-course survey was distributed via email upon registration and could be completed up to the start of the first session. The post-course survey was distributed during the final session and followed up by email the next day.

The *mean confidence score* per questionnaire-item were calculated for each of the 25 items. Subsequently, a *mean overall confidence score* was calculated for each of the four timepoints (i.e. pre-2023, post-2023, pre-2024 and post-2024) by creating the mean confidence value of each participant and then calculating the mean of all the participants’ mean values for each of the four timepoints.

Participation in the course and the surveys was voluntary and, to ensure unbiased responses from participants, the survey was anonymous. No formal assessments (e.g., examinations) were conducted, as the course was offered outside of mandatory work hours.

### Data analysis and reporting

We performed descriptive statistics to assess changes in self-reported confidence between the pre- and post-course surveys. No p-values were calculated, since a longitudinal comparison of participant’s answers was not possible due to the anonymity of survey participation.

## Results

An interdisciplinary, multicentre online teaching course on antimicrobial stewardship (AMS) was conducted in two consecutive cycles, comprising eight sessions each: from October 5 to December 7, 2023, and from May 7 to June 25, 2024. While the session topics varied slightly between the two years, the overall content remained consistent (Fig. 2A).

Attendance per session ranged from 120 (first session) to 70 (final session) in 2023 and from 140 (first session) to 64 (final session) in 2024. The average number of participants per session across both years was 99 (Fig. 2B). Participants from all three participating institutions attended the course. In both years, the majority of attendees were physicians, while only few pharmacists and students of medicine or pharmacology attended (data not shown). >90% of persons who registered for the course in 2024 had not registered for the course in 2023; detailed demographic data were not collected.

To evaluate Kirkpatrick Level 1 (Reaction), each session of the eight-session course was evaluated by participants using a five-point scale based on the German school grading system (1 = very good, 5 = inadequate). The *mean session rating scores* for each of the three items content, didactics, and applicability (calculated per item per session) revealed largely good (2) to very good (1) ratings. The only notable exception was session 3 in 2023, which received worse ratings (*mean session rating scores* of 1.86, 2.14, and 2.14, respectively) and was subsequently restructured and integrated into session 1 in 2024. The *mean session rating scores* are demonstrated in numeric format in Fig. 2A and in graphical format in Fig. 2C.

The *mean overall session scores* for content, didactics, and applicability were 1.40, 1.57, and 1.52, in 2023 and 1.61, 1.67, and 1.57 in 2024, respectively.

To access the participants’ self-reported confidence in managing antibiotic therapy (i.e. Kirkpatrick Level 2, Learning), a pre–post survey was administered that used a 7-point scale where lower scores indicated greater perceived preparedness (1 = very well prepared, 4 = sufficiently prepared, 7 = not prepared at all).

Survey response rates differed markedly between the years and were lower for post-course assessments. In 2023, 72 participants completed the pre-course survey and 34 the post-course survey. In 2024, 193 pre-course and 38 post-course responses were recorded.

**Fig. 3:**
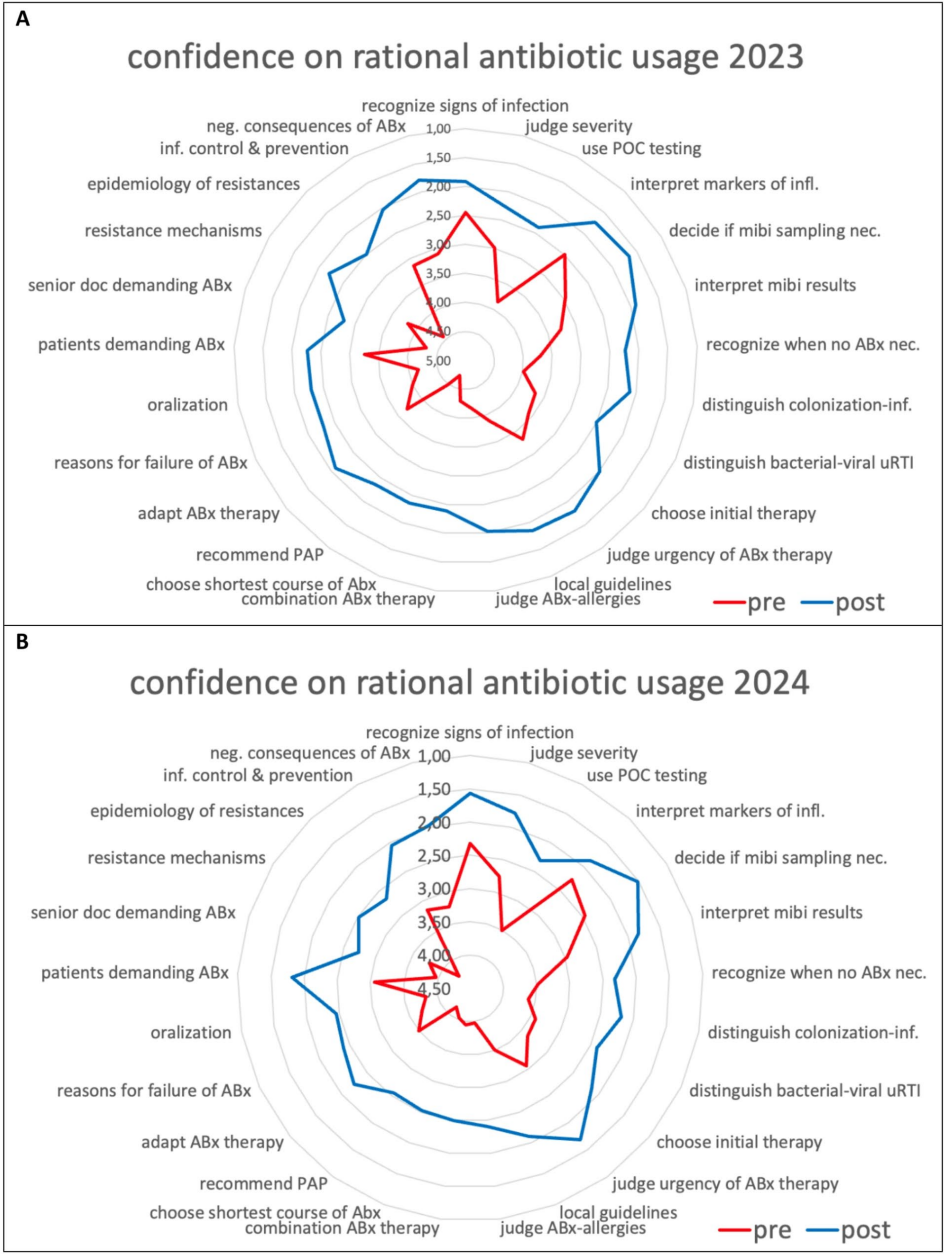
Pre- and post-course self-assessment of participants’ confidence in managing antibiotic therapy. Displayed are umbrella-charts regarding participants’ confidence in handling antibiotic agents before (“pre”, red) and after (“post”, blue) the course in 2023 (**A**) and 2024 (**B**). Participants were asked to rate their confidence regarding 25 items (details in supplement) on a seven-stepped scale where lower scores indicate greater perceived preparedness (1 = very well prepared; 4 = sufficiently prepared; 7 = not prepared at all). Displayed are the *mean confidence scores* per questionnaire-item. ABx: antibiotics, POC: point-of-care, mibi: microbiological, uRTI: upper respiratory tract infection, PAP: perioperative antibiotic prophylaxis, nec: necessary, inf: infection, neg: negative

The *mean confidence score* per questionnaire-item improved from the pre- (before the eight-session course) to the post-course survey (after the eight-session course) in 2023 and 2024. A graphical presentation of the *mean confidence score* per questionnaire-item is displayed in Fig. 3, numerical details can be found in the supplement.

In 2023, the *mean overall confidence score* improved from 3.67 (95% CI: 3.43–3.90) to 2.17 (95% CI: 1.85–2.48) with a mean difference of 1.52 (95% CI: 1.33–1.71). In 2024, it improved from 3.40 (95% CI: 3.25–3.55) to 2.19 (95% CI: 1.99–2.38) with a mean difference of 1.23 (95% CI: 1.12–1.34). The highest difference between the pre- and post-course survey could be observed in the following three items: “assessment of antibiotic allergies” (both years; increase by 2.14 in 2023 and 1.57 in 2024), “recommendation of perioperative prophylaxis” (both years; increase by 2.13 and 1.60), “start an antibiotic therapy in accordance to local guidelines” (2023; increase by 2.03) and “local epidemiology of resistances” (2024; increase by 1.59).

Comparative analysis of learning outcomes between medicine and pharmacy students was not possible due to anonymized survey responses, but participants from both professions consistently reported that the interdisciplinary format enhanced their learning experience and fostered appreciation for interprofessional collaboration.

## Discussion

This study examined the effects of an interdisciplinary, multicentre online teaching course on antimicrobial stewardship (AMS) on healthcare workers’ (HCWs) self-perceived confidence in managing antibiotic therapy. In both iterations of the course, participants reported increased confidence across all surveyed domains. The biggest increases were observed in the items on antibiotic allergies, perioperative prophylaxis, local guidelines and epidemiology of resistances.

Our findings are concordant with prior evaluations of AMS education. In primary care, internet-based training in communication skills and/or CRP use reduced antibiotic prescribing in multinational cluster RCTs [14] and the effect on prescribing persisted long-term [15]. In hospitals, a review concluded that stewardship interventions — including educational/behavior-change components — safely reduce unnecessary antibiotic use and improve guideline adherence [16]. Against this backdrop, an improvement in self-efficacy after our eight-hour, interprofessional course is expected under Adult Learning Theory (practice-centered, immediately relevant content) and Social Cognitive Theory (mastery experiences, modelling, and feedback increases self-efficacy as a proximal determinant of behavior).

Historically, Germany has had a shortage of formally trained infectious disease specialists, with a board-certified specialty (“Facharzt”) only introduced in 2021. Given these limitations in personnel resources, the implementation of a collaborative, inter-institutional training program represents a practical and scalable approach to supporting AMS education. Online teaching platforms offer flexible, accessible opportunities to disseminate key AMS principles across professional groups, regardless of location or institutional capacity.

While we assessed Kirkpatrick Levels 1–2 (Reaction and Learning/Self-efficacy) [17], the abovementioned trials show that targeted AMS education can translate into behavior change (lower antibiotic prescribing after clinician training [14], reduction of antibiotic use in hospitals after stewardship interventions [16]). Thus, our observed gains in confidence represent a plausible proximal step toward improved prescribing, but require confirmation with Level-3 metrics. Future studies could incorporate behavioral endpoints — e.g., guideline-concordant initial choice, duration of therapy, antibiotic-free days — and, where feasible, compare these before and after participation.

The interdisciplinary nature of the course — integrating pharmacists, infectious disease specialists, and microbiologists — enabled a more holistic understanding of antimicrobial therapy. In our study, direct comparison of quantitative learning outcomes between medicine and pharmacy practitioners/students was not feasible due to survey anonymization. However, this collaborative approach is aligned with current evidence on successful AMS interventions and is transferable to other clinical education settings. Interprofessional education initiatives – such as joint case-based learning – can lead to improved collaborative competencies, deeper understanding of roles, and greater satisfaction among both groups [18].

However, this study has several limitations. First, session attendance declined over time in both course iterations. While this phenomenon is commonly reported in longitudinal educational formats [8], it may be influenced by scheduling constraints, workload, or competing priorities among participants. Notably, our course was limited to eight sessions to address this issue, drawing on previous experiences where higher dropout rates were observed in longer courses.

Second, while a validated survey instrument was used to assess learners’ confidence [13], self-reporting bias in survey responses might have occurred. We tried to mitigate this by anonymous survey responses; however, this choice left us unable to determine volunteer bias as we could not match survey responses with demographic data.

Third, the study had no control group, as randomization was not feasible.

Fourth, response rates were lower than anticipated—particularly for the post-course survey, which likely also contributed to volunteer bias. The decision to anonymize responses was made to encourage honest feedback and avoid potential discomfort in interprofessional settings. However, anonymity may have reduced participants’ perceived obligation to complete the survey. For future iterations, strategies to increase engagement — such as reminders, incentives, or anonymous tracking — will be considered.

## Conclusion

This interdisciplinary AMS teaching course effectively enhanced participants’ confidence in responsible antibiotic prescribing, most effectively in the areas of antibiotic allergies, perioperative prophylaxis, use of local antibiotic guidelines and epidemiology of resistances. Key strengths included its interprofessional design, integration of pharmacological fundamentals with case-based clinical learning, and the use of structured, consistent teaching methods. Regular evaluation of both didactic approaches and learner outcomes is essential to continuously improve the course.

Challenges such as participant dropout and low survey completion rates will be addressed in the upcoming 2025 course cycle. With further refinement, this course format may serve as a sustainable contribution to local AMS efforts and support the broader goal of combating antimicrobial resistance through targeted education.

## Supplementary Information

Supplementary Material 1

## Data Availability

The datasets used and analysed in the study are available from the corresponding author on reasonable request.

## Abbreviations

AMR: Antimicrobial resistance
HCW: Healthcare workers
AMS: Antimicrobial stewardship
PBL: Problem-based learning
OC: Organizing committee
UKSH: University Hospital Schleswig-Holstein
UKE: University Medical Center Hamburg-Eppendorf
REDCap: Research Electronic Data Capture
