# Supplementary material for: Improving antibiotic stewardship skills through interdisciplinary online education: a multicenter teaching intervention for physicians and pharmacists
Source: BMC Med Educ. 2025 Dec 17;26:110. doi: 10.1186/s12909-025-08348-4 (PMC12821917; doi:10.1186/s12909-025-08348-4)
Supplement: Supplementary file 1 — Supplementary Material 1 [file 12909_2025_8348_MOESM1_ESM.docx]

**Supplementary appendix to**

Improving Antibiotic Stewardship Skills through Interdisciplinary Online Education:

A Multicenter Teaching Intervention for Physicians and Pharmacists

Till Koch^1,2,3^, Johannes Jochum^1,4,5^, Annika van der Linde^1^, Friederike Jahn^6^, Anika Neubert^7^, Evelyn Kramme^3,8,9^†, Anette Friedrichs^10,11^†

^1^Antibiotic Stewardship Team, Pharmacy of the University Medical Center Hamburg-Eppendorf, Hamburg, Germany

^2^ 3rd Medical Clinic for Pneumology, Infectious Diseases and Oncology, Klinikum Itzehoe, Itzehoe, Germany

^3^ German Center for Infection Research (DZIF), Partner Site Hamburg-Lübeck-Borstel-Riems, Hamburg, Germany

^4^ 1st Department of Medicine, University Medical Center Hamburg-Eppendorf, Hamburg, Germany

^5^ Center for Tropical Medicine, Bernhard Nocht Institute for Tropical Medicine, Hamburg, Germany

^6^ Antibiotic Stewardship Team, Pharmacy of the University Medical Center Schleswig-Holstein, Campus Kiel, Kiel, Germany

^7^ Antibiotic Stewardship Team, Pharmacy of the University Medical Center Schleswig-Holstein, Campus Lübeck, Lübeck, Germany

^8^ Antibiotic Stewardship Team, Commercial Management, University Medical Center Schleswig-Holstein, Campus Lübeck, Lübeck, Germany

^9^ Department of Infectious Diseases, University Medical Center Schleswig-Holstein, Campus Lübeck, Lübeck, Germany

^10^ Antibiotic Stewardship Team, Commercial Management, University Medical Center Schleswig-Holstein, Campus Kiel, Kiel, Germany

^11^ 1st Department of Medicine, University Medical Center Schleswig-Holstein, Campus Kiel, Kiel, Germany

†both authors contributed equally

Corresponding author:

Till Koch

Robert-Koch-Str.2, 25524 Itzehoe

[t.koch@kh-itzehoe.de](mailto:t.koch@kh-itzehoe.de)

tikoch@protonmail.com

Tel +49 152 5913-6108

Fax +49 4821 772-2309

﻿ORCID: 0000-0002-0140-2808

**Content:**

1. Comment regarding removal of two questions
2. Original survey (English)
3. Adapted survey (German)
4. Example slides
5. Questions used to set the learning goals for each session and structure its content
6. *Mean confidence score* per questionnaire-item

**1. Comment regarding removal of two questions**

Specifically, the two excluded questions were addressing antimicrobial stewardship programs (“…to measure/audit antibiotic use in a clinical setting, and to interpret the results of such studies”; “…to work within the multidisciplinary team in managing antibiotic use in hospitals”), which was not covered in the course.

**2. Original survey (English)**

Available online at [https://academic.oup.com/jac/article/73/8/2236/4994358#supplementary-data](https://academic.oup.com/jac/article/73/8/2236/4994358#supplementary-data), supplement to article <https://academic.oup.com/jac/article-pdf/73/8/2236/25178723/dky150.pdf>


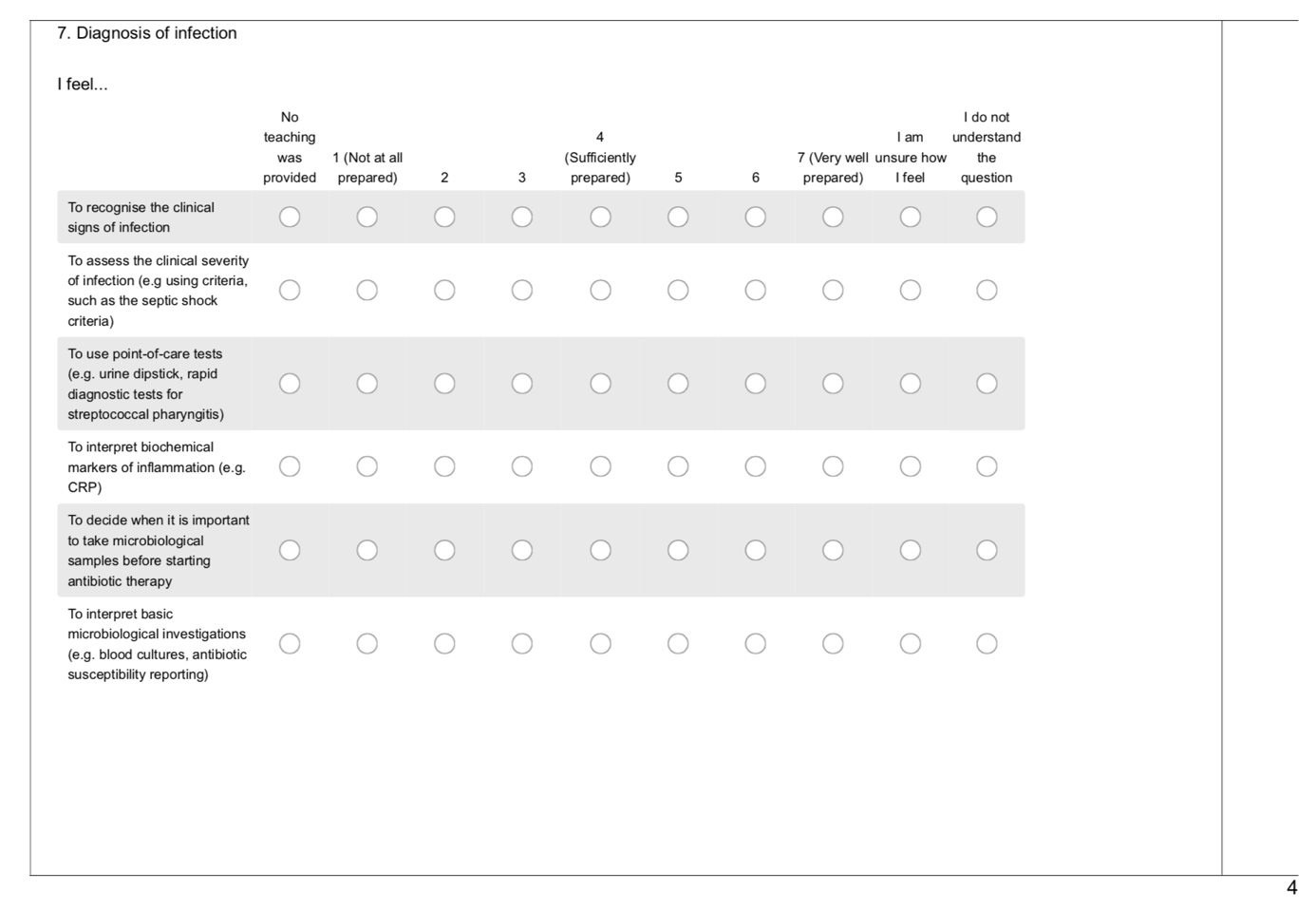


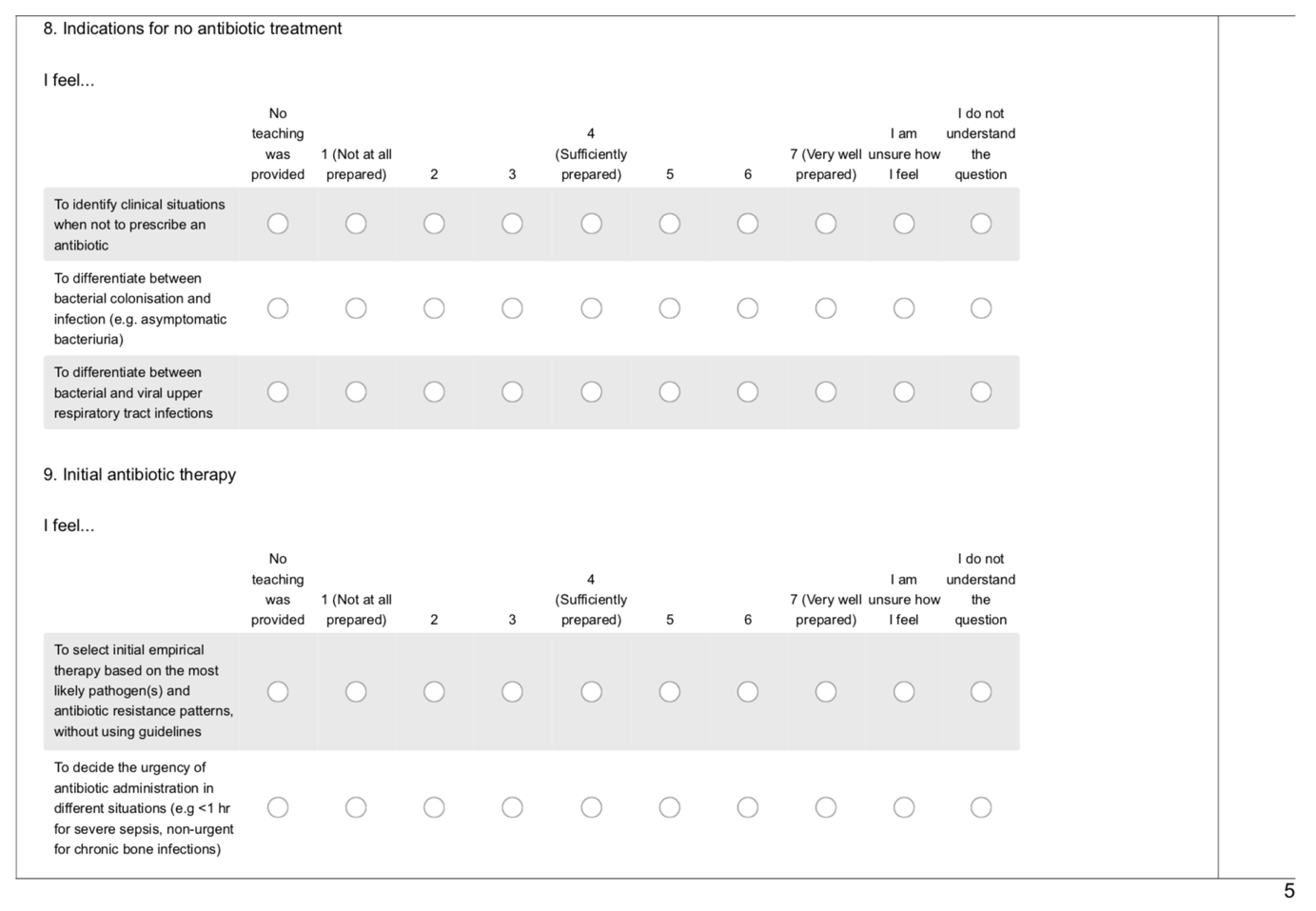


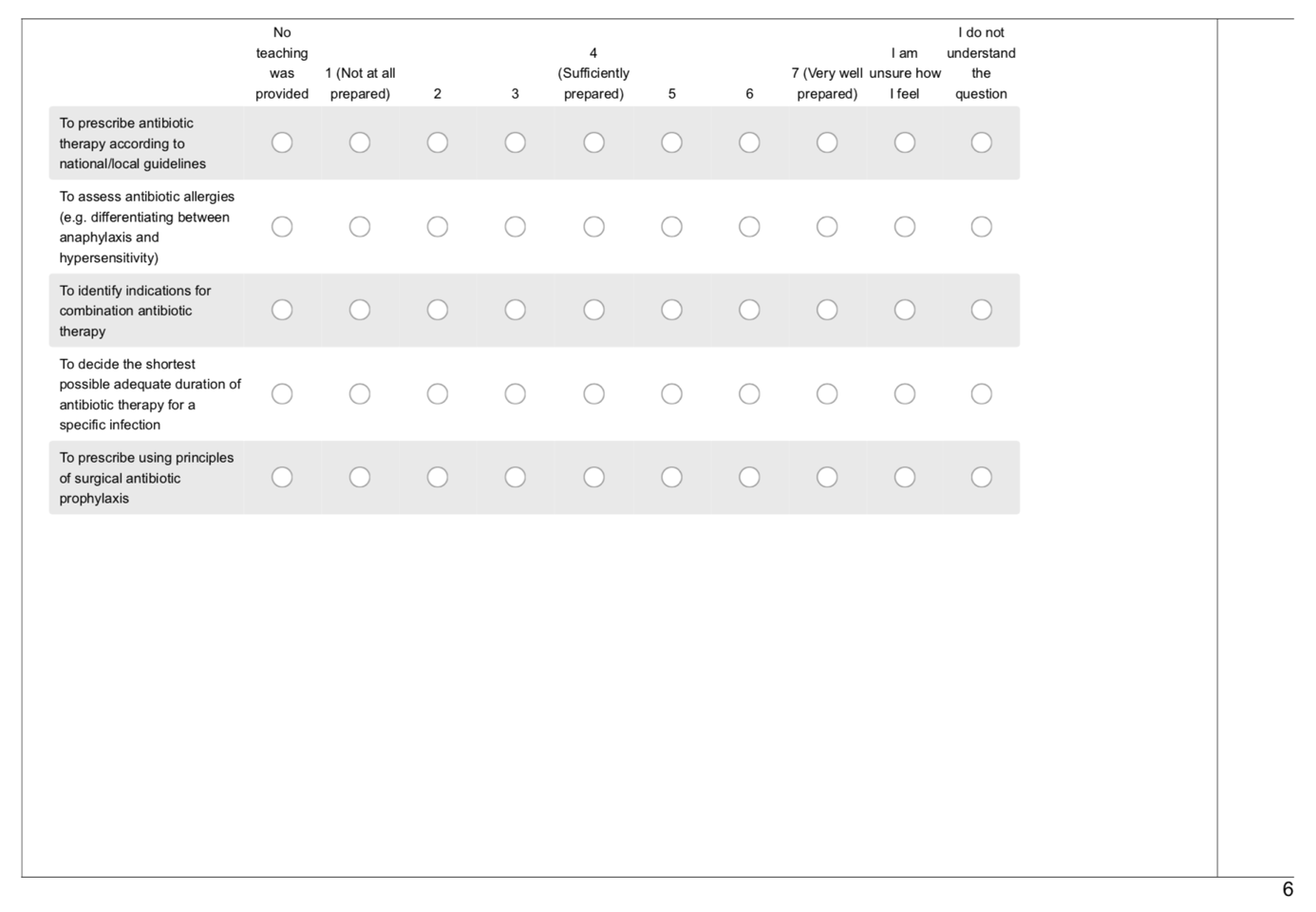


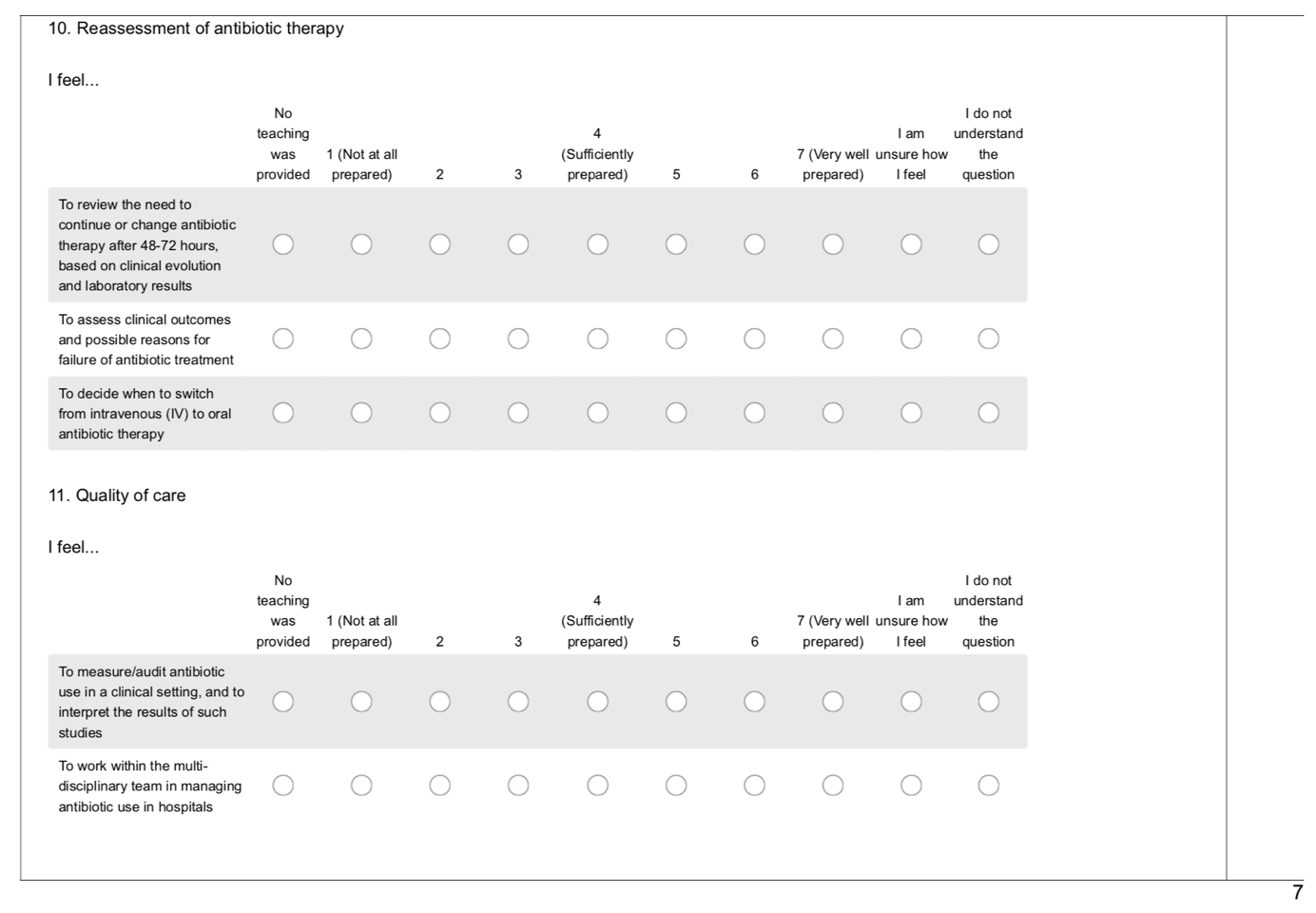


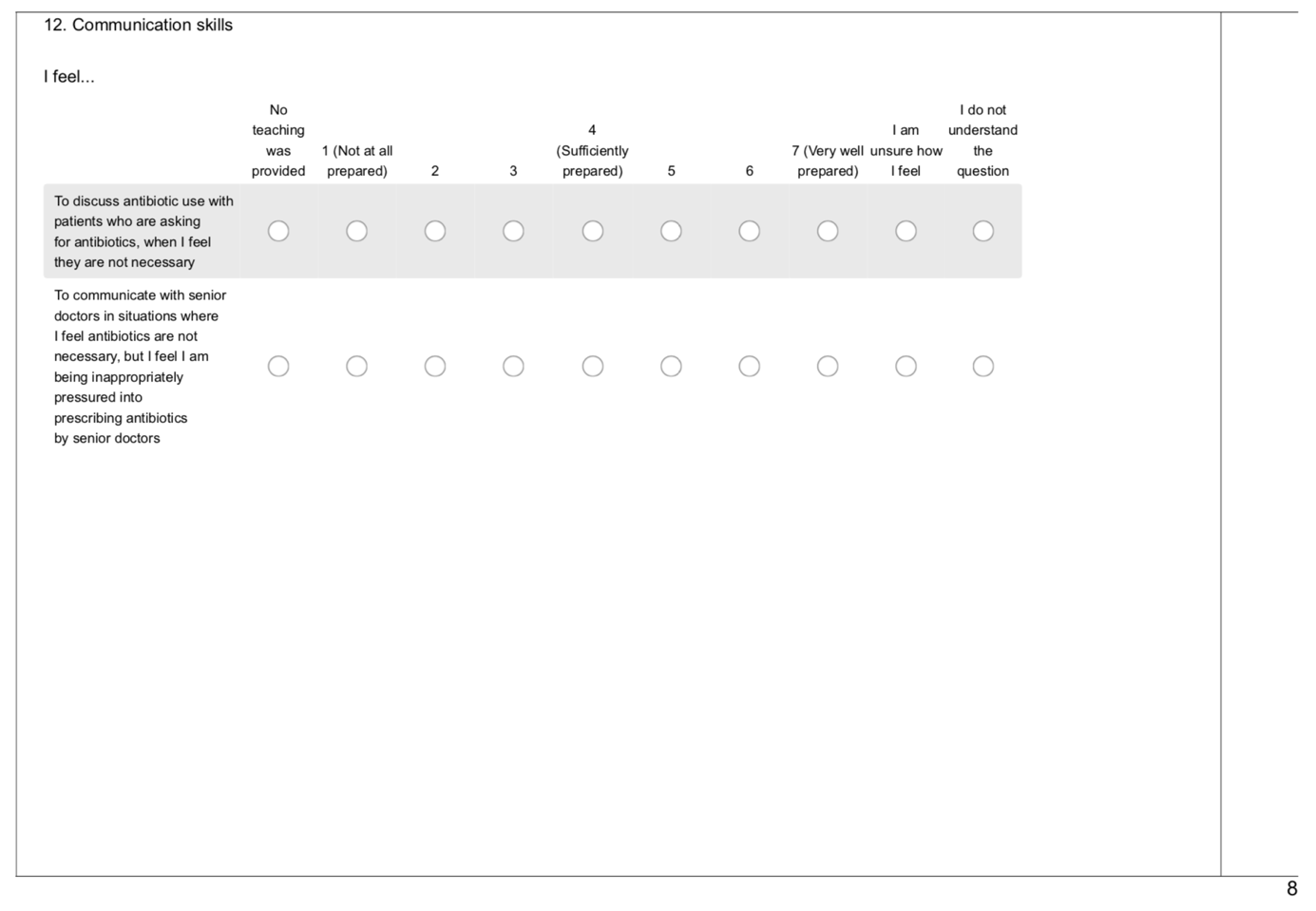


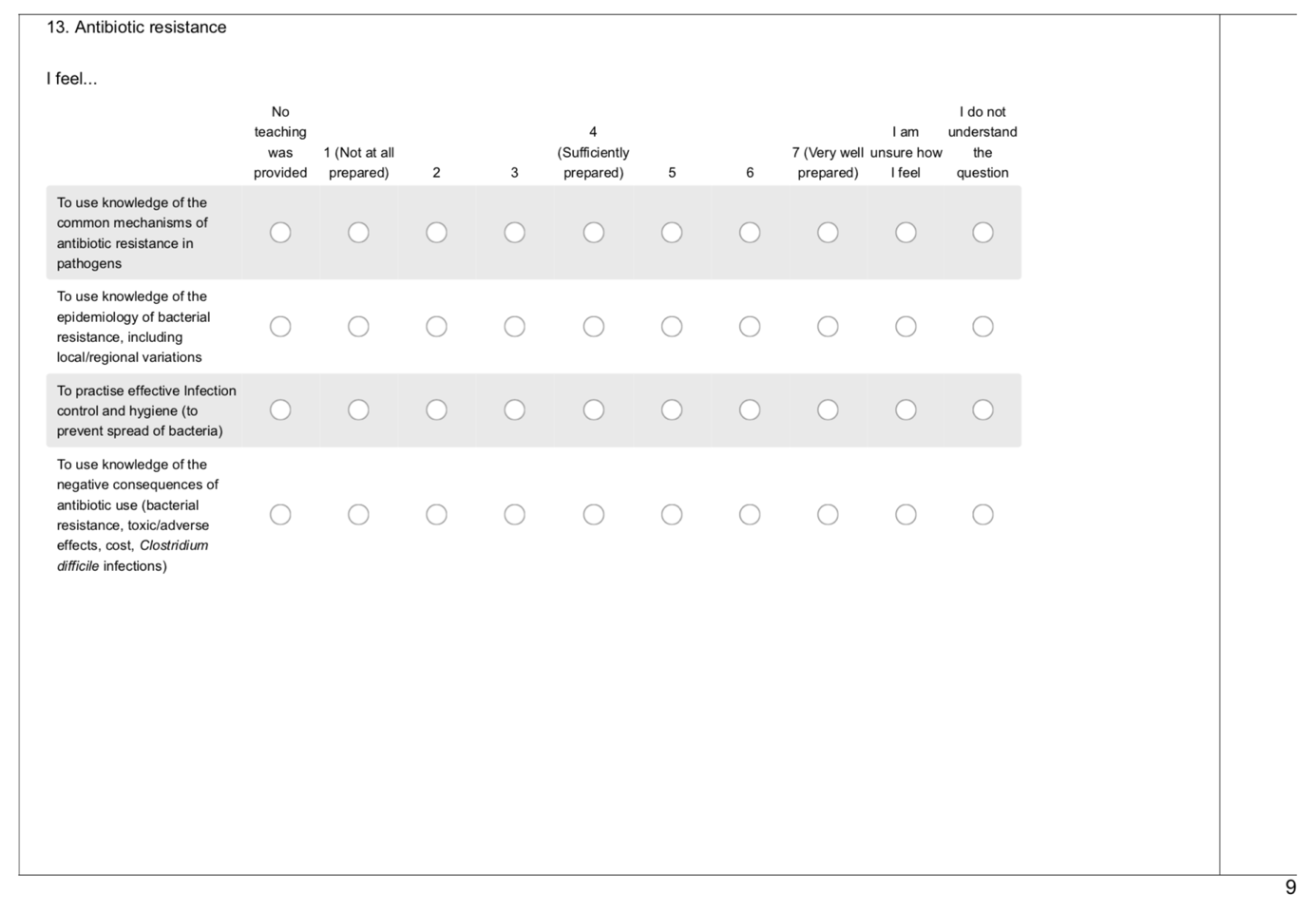


**3. Adapted survey (German)**

**Umfrage Antiinfektivakurs Nord**

7-Stufige Skala von 1 (Ich fühle mich gar nicht vorbereitet) über 4 (ich fühle mich ausreichend vorbereitet) zu 7 (ich fühle mich sehr gut vorbereitet)

**A)    Diagnose einer Infektion:**

Wie gut fühle ich mich vorbereitet…

1)    die klinischen Anzeichen einer Infektion zu erkennen

2)    den klinischen Schweregrad einer Infektion zu beurteilen (z. B. anhand von Kriterien für septischen Schock, CRB65)

3)    Point-of-Care-Tests sinnvoll einzusetzen (z. B. Urintest, Schnelltests zur Diagnose von Streptokokken-Pharyngitis)

4)    laborchemische Entzündungsmarker zu interpretieren (z. B. CRP)

5)    zu entscheiden, wann die Entnahme von mikrobiologischen Proben VOR Beginn der Antibiotikatherapie erfolgen sollte?

6)    die Ergebnisse mikrobiologischer Untersuchungen zu interpretieren (z. B. Blutkulturen, Antibiotika-Resistogramme)

**B) Verzicht auf antibiotische Therapie**

Wie gut fühle ich mich vorbereitet…

7)    klinische Situationen zu erkennen, in denen kein Antibiotikum verschrieben werden sollte

8)    zwischen bakterieller Besiedlung und Infektion zu unterscheiden (z. B. asymptomatische Bakteriurie)

9)    zwischen bakteriellen und viralen Infektionen der oberen Atemwege zu differenzieren

**C) Initiale antibiotische Therapie**

Wie gut fühle ich mich vorbereitet…

10)  eine initiale empirische Therapie auf der Grundlage der wahrscheinlichsten Erreger und Antibiotikaresistenzmuster auszuwählen

11)  die Dringlichkeit einer Antibiotikagabe in verschiedenen Situationen einschätzen zu können (z. B. <1 Stunde bei schwerer Sepsis, nicht dringlich bei chronischen Knocheninfektionen)

12)  Antibiotikatherapien gemäß den nationalen/lokalen Leitlinien zu beginnen

13)  Antibiotika-Allergien zu beurteilen (z. B. Unterscheidung zwischen leichten und schweren Formen der Allergie) diese Differenzierung finde ich komisch. Überempfindloichkeit ist doch das deutsche Wort für Allergie? Und Anaphylaxie wäre eine starke /schwere Allergie

14)  Indikationen für eine Antibiotika-Kombinationstherapie zu erkennen

15)  die kürzest mögliche angemessene Dauer der Antibiotikatherapie bei einer bestimmten Infektion zu bestimmen

16)  eine Perioperative Antibiotikaprophylaxe (PAP) zu empfehlen

**D) Re-evaluation der Antibiotikatherapie**

Wie gut fühle ich mich vorbereitet…

17)  die Notwendigkeit einer Fortsetzung oder Änderung der Antibiotikatherapie nach 48-72 Stunden auf der Grundlage der klinischen Entwicklung und der Laborergebnisse beurteilen zu können

18)  die klinischen Ergebnisse und mögliche Gründe für das Scheitern einer Antibiotikabehandlung bewerten zu können

19)  zu entscheiden, wann von einer intravenösen (IV) auf eine orale Antibiotikatherapie umgestellt werden sollte

**E) Kommunikative Kompetenz**

Wie gut fühle ich mich vorbereitet…

20)  den Einsatz von Antibiotika mit Patient:Innen zu besprechen, die gerne ein Antibiotikum hätten, während ich ein Antibiotikum nicht für notwendig halte

21)  mit leitenden Ärzt:Innen in Situationen zu kommunizieren, in denen ich Antibiotika nicht für notwendig halte, aber von leitenden Ärzt:Innen in unangemessener Weise zur Verschreibung von Antibiotika gedrängt werde

**F) Antibiotikaresistenz**

Wie gut fühle ich mich vorbereitet…

22)    die allgemeinen Mechanismen der Antibiotikaresistenz bei Krankheitserregern zu kennen

23)    Kenntnisse über die Epidemiologie der bakteriellen Resistenzen, einschließlich lokaler/regionaler Unterschiede, nutzen zu können

24)    effektive Infektionskontrolle und Hygienemaßnahmen durchzuführen (um die Verbreitung von Bakterien zu verhindern)

25)    das Wissen um die negativen Folgen des Einsatzes von Antibiotika im klinischen Alltag anwenden zu können (bakterielle Resistenz, toxische/unerwünschte Wirkungen, Kosten, Clostridioides difficile-Infektionen)

**4. Example slides from the course *Antiinfektivakurs Nord* held in 2024**

Specifically, here are some slides from the second session that covered penicillins and pneumonia.


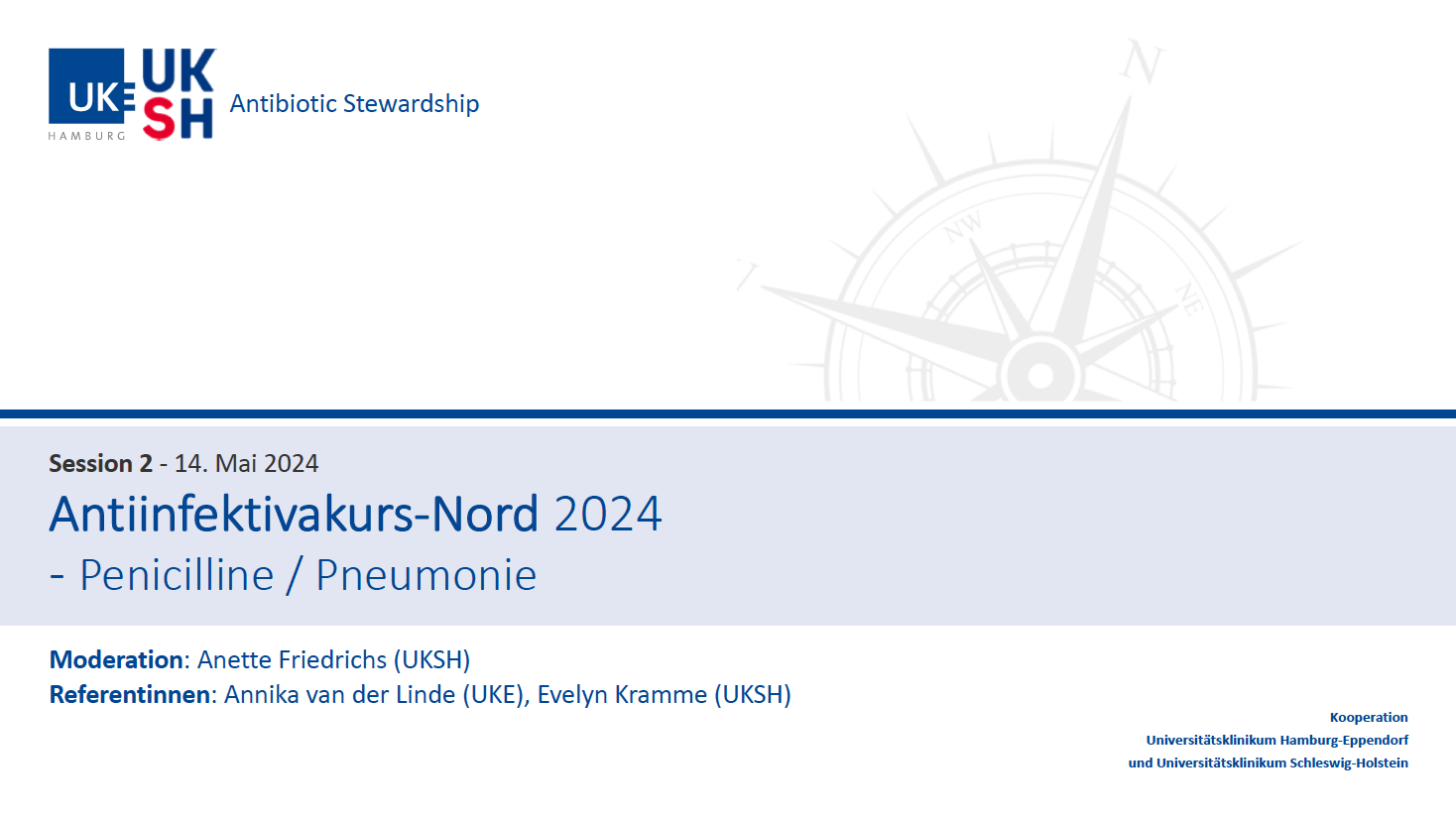

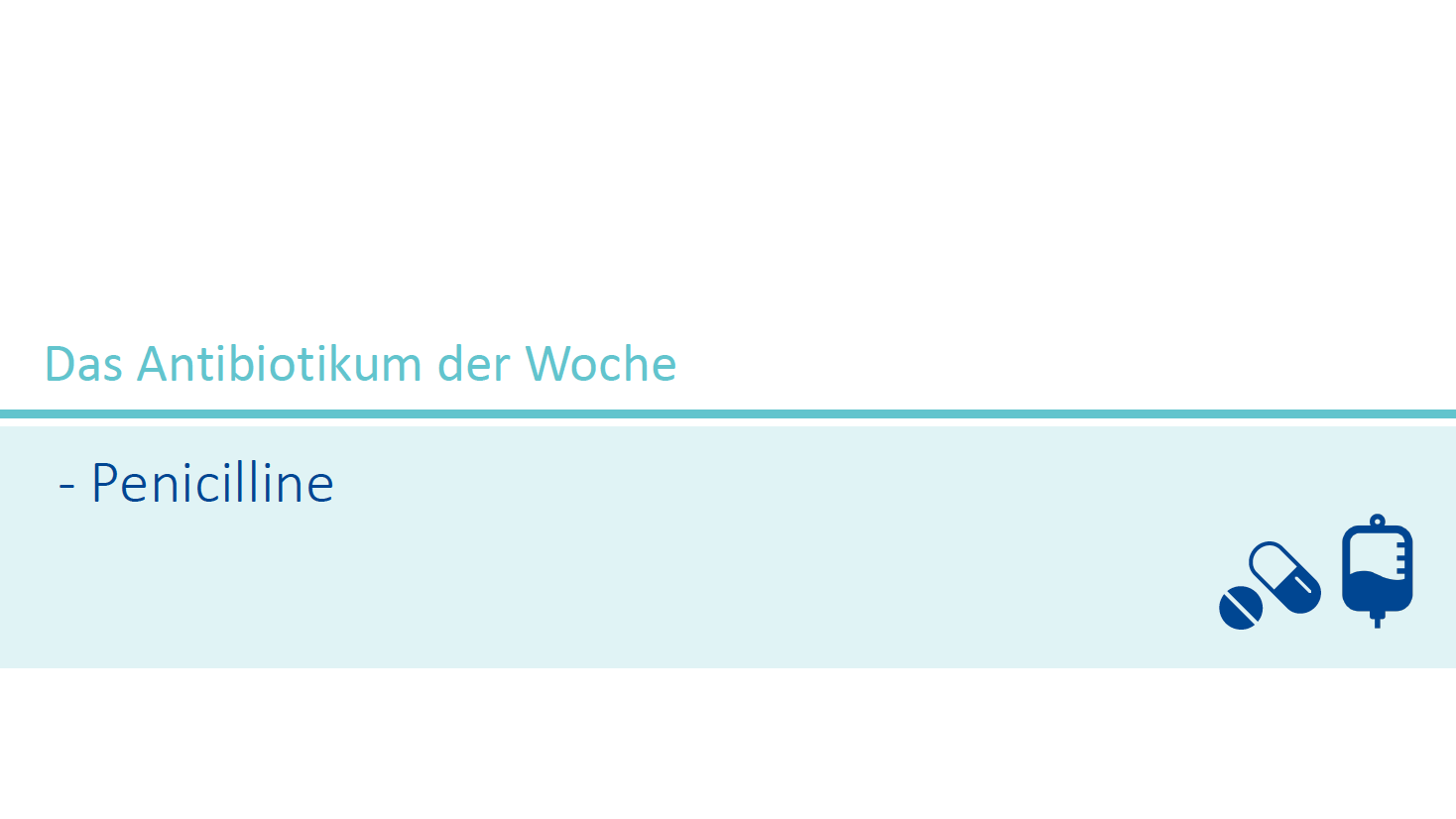

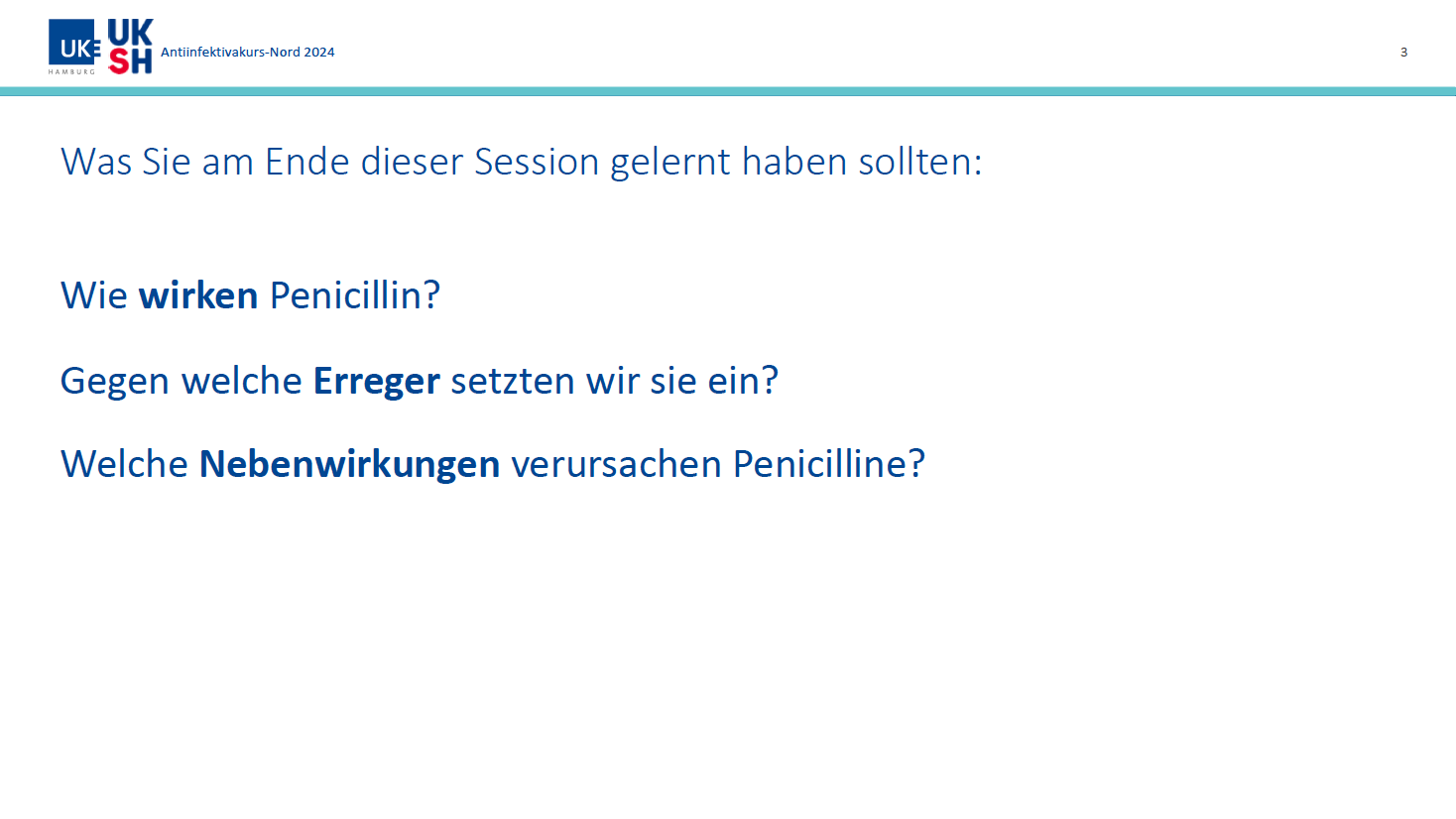

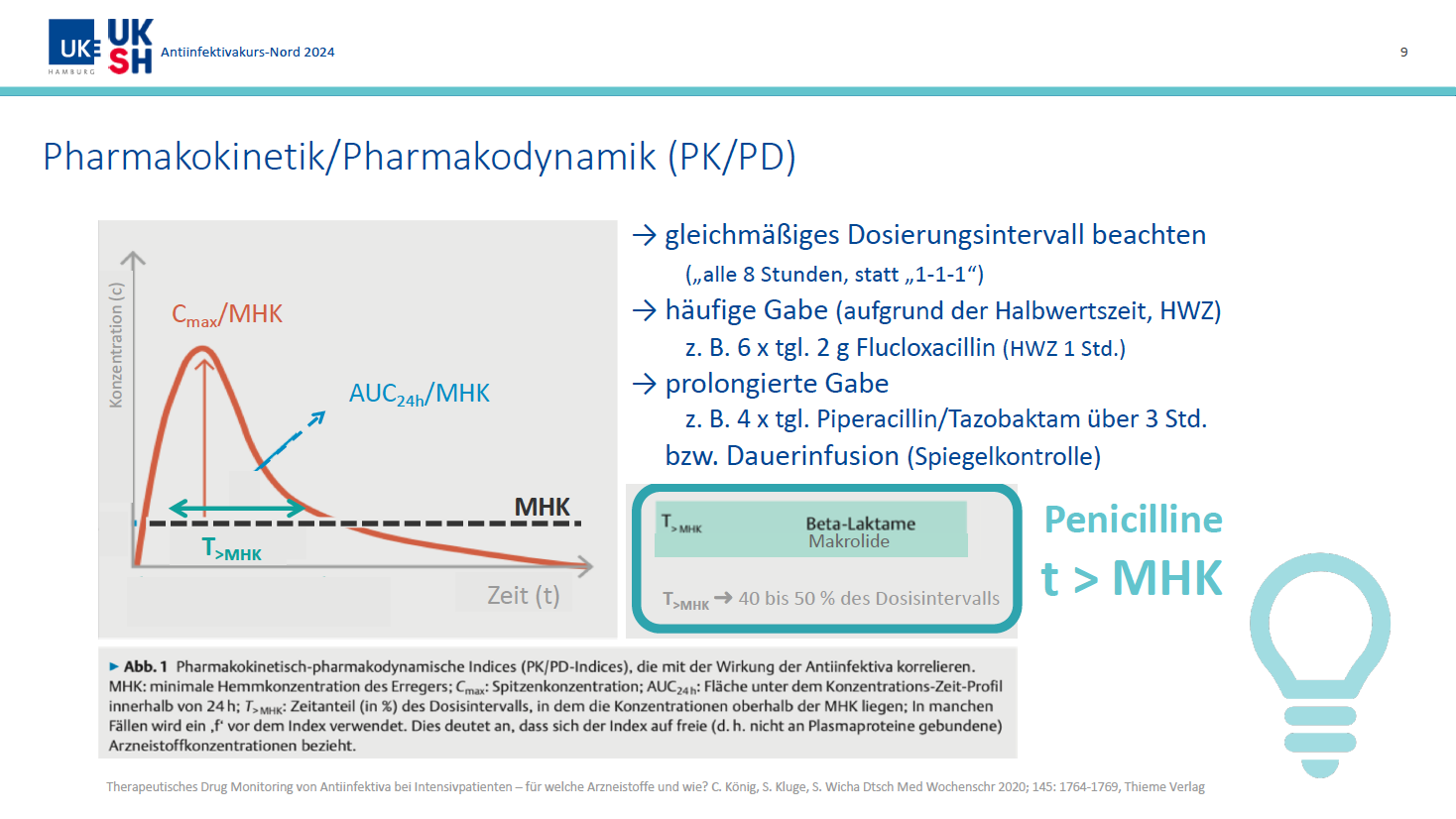

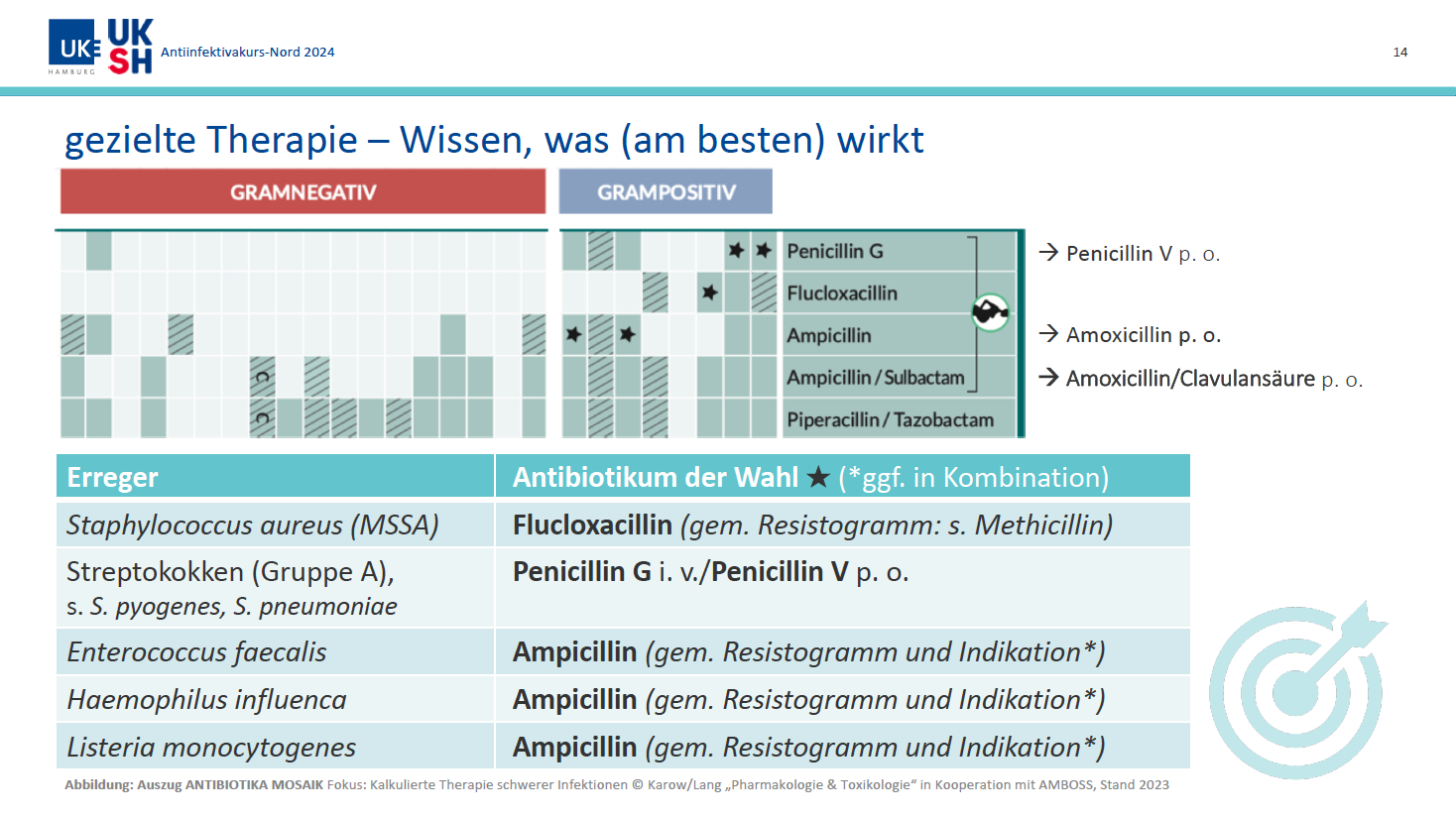

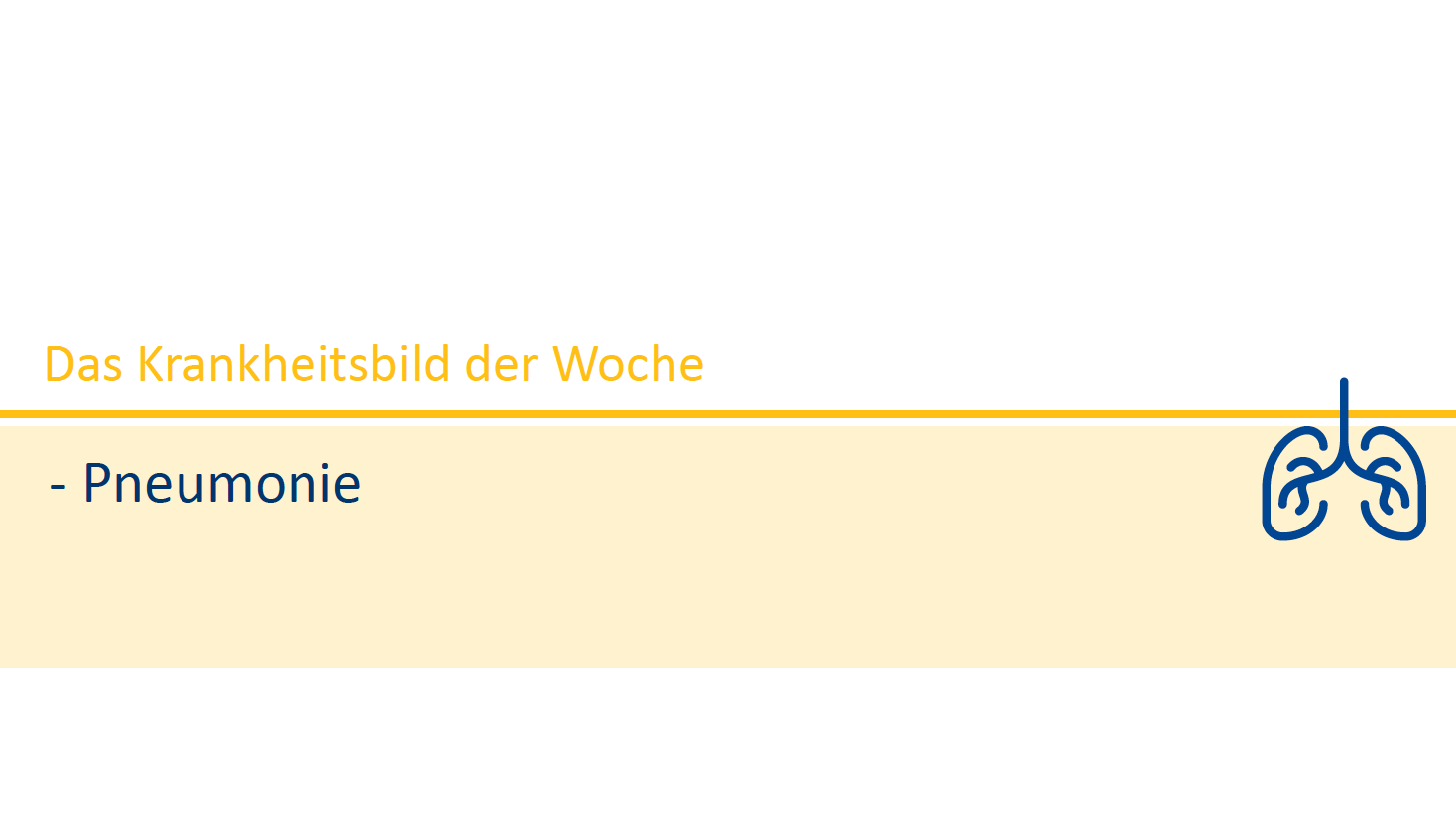

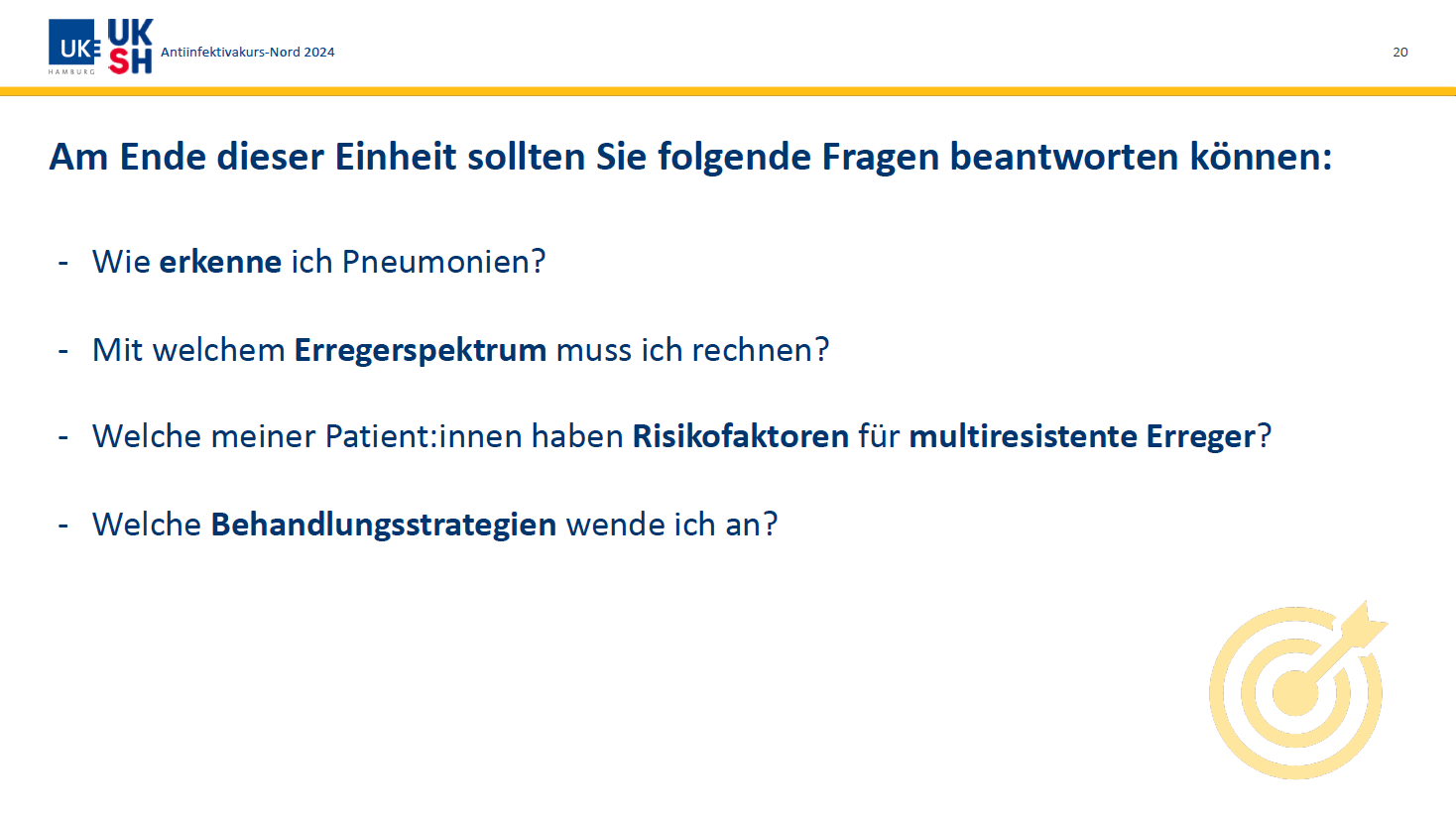

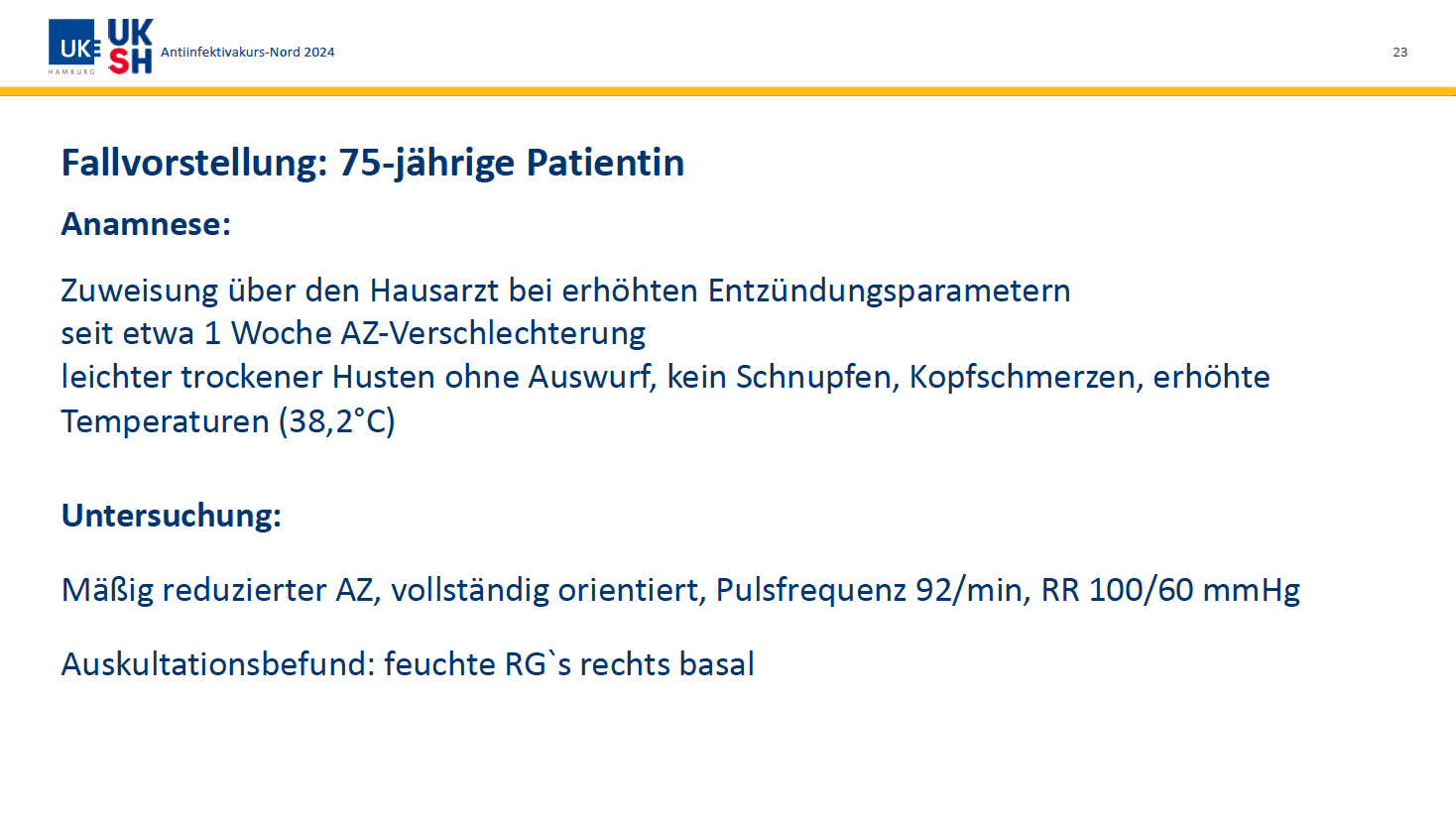

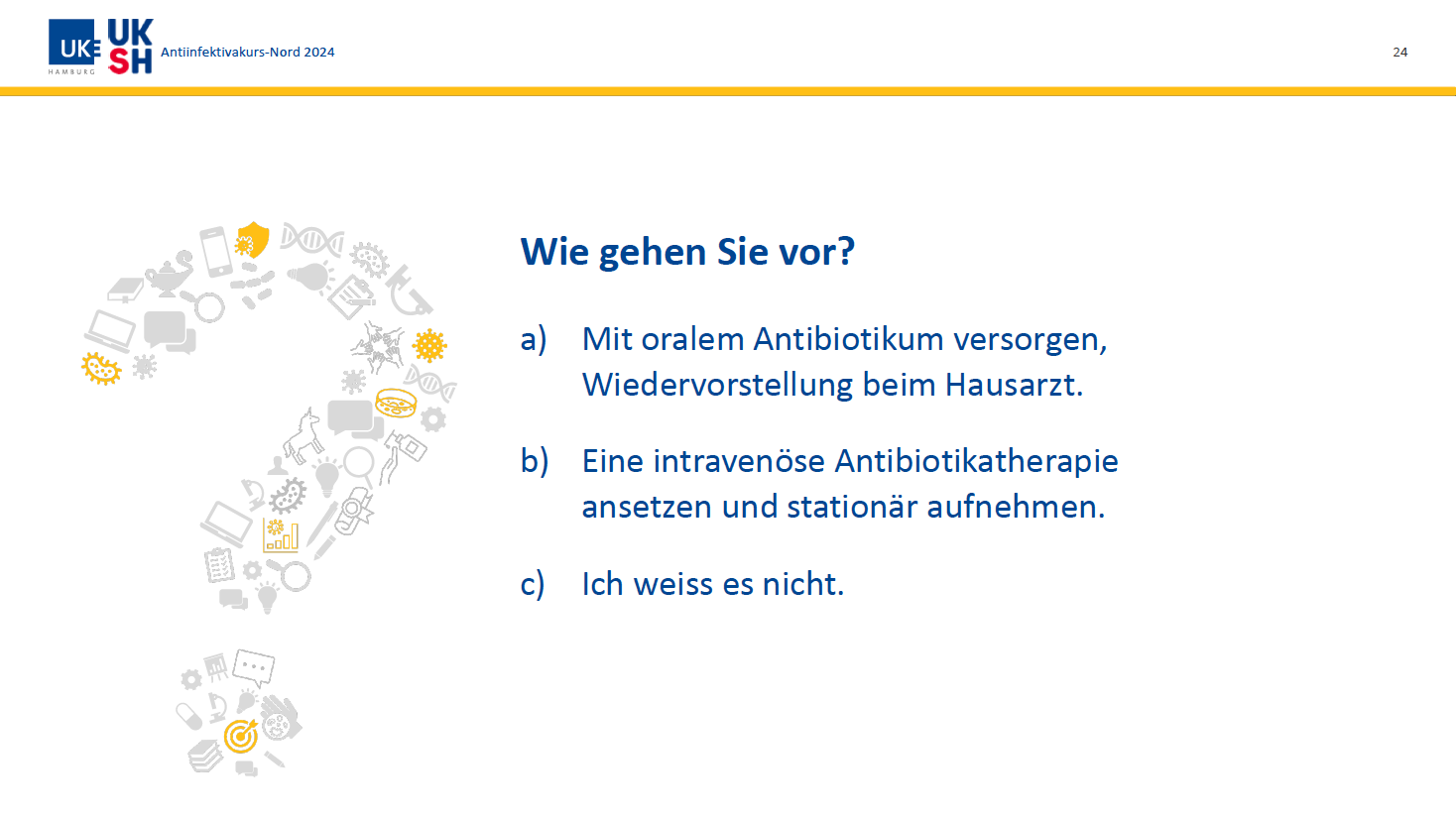

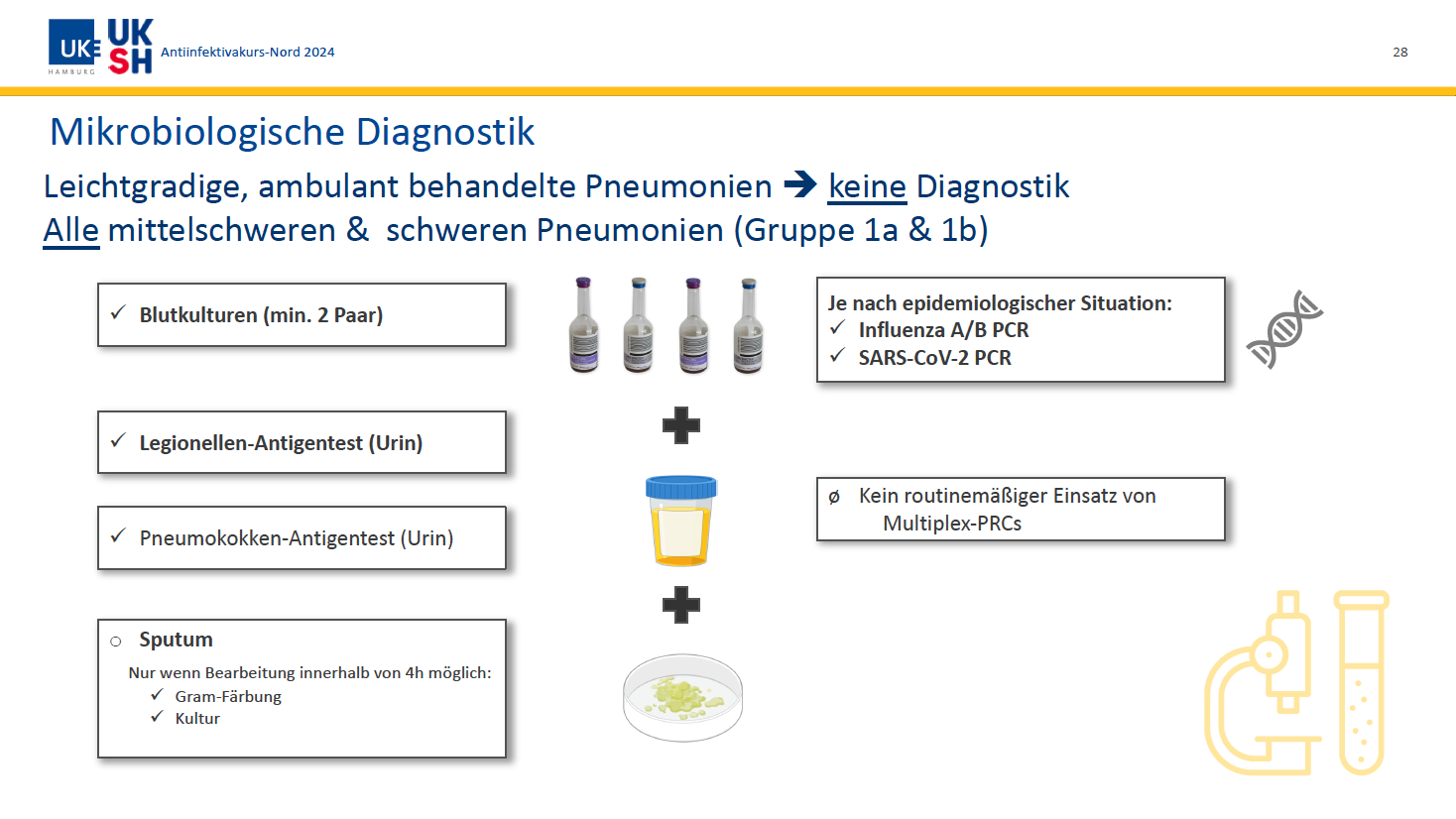

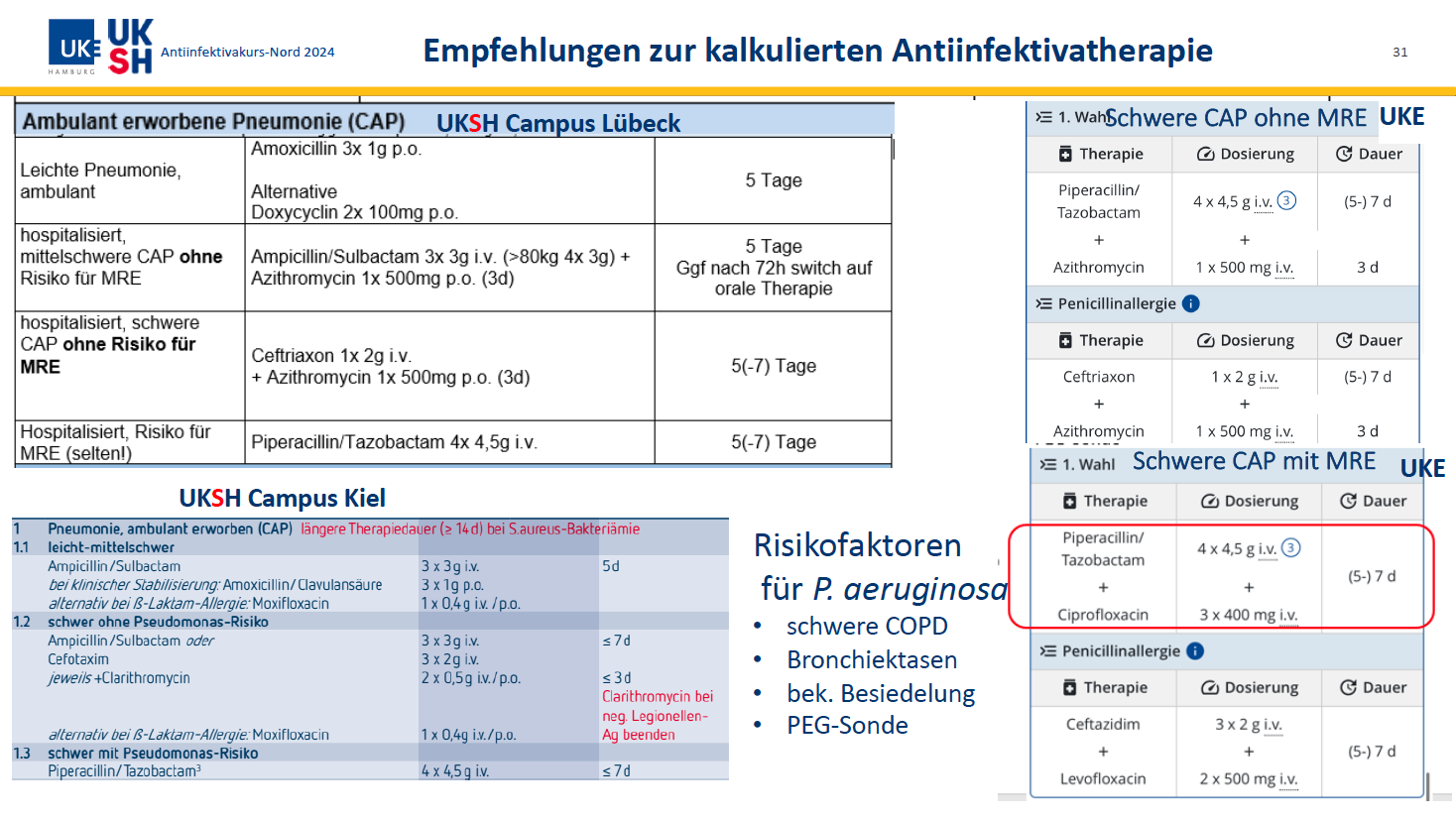

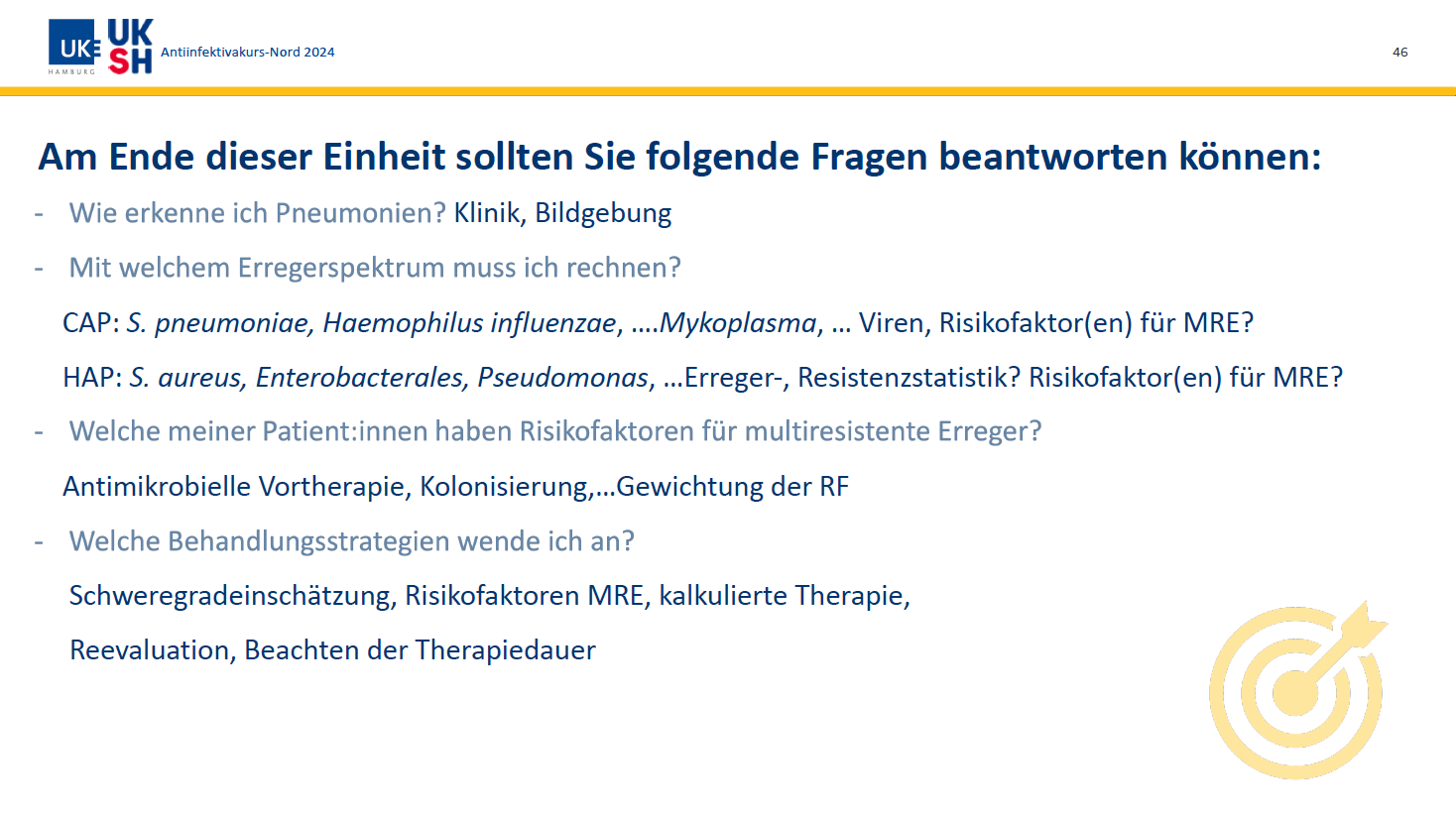


**5. Questions used to set the learning goals for each session and structure its content**

| **Session No (Topics)** | **Questions (german)** | **Questions (English translation)** |
| --- | --- | --- |
| 1  (Antimicrobial stewardship & Microbiology) | - Was bedeutet ABS ? - Welche Antibiotika-Therapiestrategien sollten Sie anwenden? - Welche Strategien wenden Ihre ABS-Teams an? - Welche Patient:innen profitieren von infektiologischen Konsilen? | - What does ABS mean? - Which antibiotic therapy strategies should you use? - What strategies do your ABS teams use? - Which patients benefit from infectious disease consults? |
| 2  (Penicillins & Pneumonia) | - Wie wirken Penicillin? - Gegen welche Erreger setzten wir sie ein? - Welche Nebenwirkungen verursachen Penicilline? - Wie erkenne ich Pneumonien? - Mit welchem Erregerspektrum muss ich [bei der Pneumonie] rechnen? - Welche meiner Patient:innen haben Risikofaktoren für multiresistente Erreger? - Welche Behandlungsstrategien wende ich an? | - How do penicillins work? - Which pathogens do we use them against? - What side effects do penicillins cause? - How do I recognize pneumonia? - What spectrum of pathogens should I expect [in pneumonia]? - Which of my patients have risk factors for multi-resistant pathogens? - What treatment strategies should I use? |
| 3  (Cephalosporins & Perioperative prophylaxis) | - Worin unterscheiden sich die einzelnen Generationen der Cephalosporine? - Welche Cephalosporine sind zur oralen Einnahme geeignet (welche nicht)? - Welche unerwünschten Arzneimittelwirkungen sind unter Metronidazol möglich? - Was ist eine perioperative Antibiotikaprophylaxe? - Wann sollte sie gegeben werden? - Wie lange sollte sie gegeben werden? | - What are the differences between the individual generations of cephalosporins? - Which cephalosporins are suitable for oral administration (which are not)? - What adverse drug reactions are possible with metronidazole? - What is perioperative antibiotic prophylaxis? - When should it be given? - How long should it be given? |
| 4  (Vancomycin & Bloodstream infections) | - Bei welcher Substanz sind regelmäßige Spiegelkontrollen erforderlich (TDM)? - Welches Medikament wird in der Praxis höher dosiert als in der Fachinformation? - Bei welchem Präparat muss ich mit relevanten Kosten rechnen? - Wie kann bei einer S.-aureus-Blutstrominfektion das Überleben verbessert werden? - Was ist die häufigste Ursache einer „Blutkultur-negativen“ Endokarditis? - Ist bei einer S. aureus-Blutstrominfektion oder einer Endokarditis eine Oralisierung der Therapie möglich? - Wie ist das Vorgehen bei V. a. Schrittmacher-Tascheninfektion? | - Which substance requires regular level checks (TDM)? - Which medication is given in higher doses in practice than those specified in the specialist information? - For which preparation should I expect relevant costs? - How can survival be improved in the case of a S. aureus bloodstream infection? - What is the most common cause of “blood culture-negative” endocarditis? - Is oral therapy possible for S. aureus bloodstream infection or endocarditis? - What is the right management of a suspected pacemaker pocket-infection? |
| 5  (Carbapenems & Sepsis) | - Wie wirken Carbapeneme? - Welche Fixkombinationen gibt es und warum? - Wann ist die Gabe von Carbapenemen indiziert? - Wie erkenne ich eine Sepsis? - Welche Erreger lösen eine Sepsis aus? - Welches sind die wichtigsten Foci? - Wie behandle ich aus infektiologischer Sicht? | - How do carbapenems work? - What fixed combinations are available? - When is the administration of carbapenems indicated? - How do I recognize sepsis? - Which pathogens cause sepsis? - What are the most important foci? - How do I treat from an infectious diseases point of view? |
| 6  (Macrolides & Skin and soft tissue infections) | - Welches Makrolid würden Sie bevorzugt einsetzen? - Mit welcher Wechselwirkung müssen Sie bei Doxycyclin rechnen? - Wie dosieren wir Cotrimoxazol? - Was ist ein gutes „default“ Antibiotikum bei Hautinfektionen in der Klinik? - Woran erkenne ich eine nekrotisierende Hautinfektion? - Was führt häufiger zu Infektionen, Hunde- Katzen- oder Menschenbisse? | - Which macrolide would you prefer to use? - What interactions should you expect with doxycycline? - How do we dose cotrimoxazole? - What is a good “default” antibiotic for skin infections in the clinic? - How can I recognize a necrotizing skin infection? - What leads to infections more frequently, dog, cat or human bites? |
| 7  (Chinolones & Urinary tract infections) | - Wie wirken Fluorchinolone? - Was sind die pharmakokinetischen und pharmakodynamischen Charakteristika? - Welche Nebenwirkungen & Zusatzrisiken sind wichtig? - Indikationen zur Therapie - Wie diagnostiziere ich eine Harnwegsinfektion? - Wie diagnostiziere ich eine asymptomatische Bakteriurie? - Muss jede Harnwegsinfektion antibiotisch behandelt werden? | - How do fluoroquinolones work? - What are the pharmacokinetic and pharmacodynamic characteristics? - Which side effects & additional risks are important? - Indications for therapy - How do I diagnose a urinary tract infection? - How do I diagnose asymptomatic bacteriuria? - Does every urinary tract infection require antibiotic treatment? |
| 8  (Penicillin allergy & case-based wrap-up) | - Wie häufig ist eine echte Penicillinallergie? - Was kennzeichnet eine echte allergische Reaktion? - Was sind Schritte zum Delabeling einer Penicillinallergie? | - How common is a true penicillin allergy? - What characterizes a true allergic reaction? - What are steps to delabeling a penicillin allergy? |

**6. *Mean confidence score* per questionnaire-item**

| **Question** | **2023_pre** | **2023_post** | **2024_pre** | **2024_post** |
| --- | --- | --- | --- | --- |
| recognize signs of infection | 2,45 | 1,91 | 2,32 | 1,57 |
| judge severity | 3,00 | 2,26 | 2,76 | 1,78 |
| use POC testing | 3,85 | 2,38 | 3,51 | 2,31 |
| interpret markers of inflammation | 2,50 | 1,73 | 2,26 | 1,86 |
| decide if mibi sampling necessary | 2,96 | 1,66 | 2,46 | 1,51 |
| interpret mibi results | 3,27 | 1,91 | 2,96 | 1,84 |
| recognize when no ABx necessary | 3,71 | 2,24 | 3,48 | 2,32 |
| distinguish colonization-infection | 3,99 | 2,12 | 3,61 | 2,18 |
| distinguish bacterial-viral uRTI | 3,67 | 2,50 | 3,42 | 2,39 |
| choose initial therapy | 3,58 | 2,00 | 3,38 | 2,14 |
| judge urgency of ABx therapy | 3,32 | 1,79 | 3,06 | 1,68 |
| local guidelines | 3,88 | 1,85 | 3,51 | 2,11 |
| judge ABx-allergies | 4,17 | 2,03 | 3,97 | 2,41 |
| combination ABx therapy | 4,29 | 2,38 | 3,95 | 2,50 |
| choose shortest course of Abx | 4,72 | 2,35 | 4,03 | 2,53 |
| recommend PAP | 4,49 | 2,35 | 4,15 | 2,55 |
| adapt ABx therapy | 3,69 | 2,09 | 3,50 | 2,24 |
| reasons for failure of Abx | 3,99 | 2,29 | 3,70 | 2,39 |
| oralization | 4,17 | 2,29 | 3,82 | 2,45 |
| patients demanding ABx | 3,25 | 2,26 | 3,06 | 1,82 |
| senior doctors demanding ABx | 4,28 | 2,79 | 3,96 | 2,74 |
| resistance mechanisms | 3,82 | 2,21 | 3,78 | 2,51 |
| epidemiology of resistances | 4,44 | 2,50 | 4,25 | 2,66 |
| infection control & prevention | 3,14 | 2,03 | 3,15 | 2,05 |
| negative consequences of ABx | 3,10 | 1,79 | 3,23 | 1,97 |

**7. Summary of course content in 2024**

A brief summary of each session’s content of the 2024 course is as follows:

**Session 1** (Antimicrobial stewardship & Microbiology):

- Antimicrobial stewardship: Strategies, local guidelines, duration of therapy.
- Microbiology: importance of adequate pre-analytic quality, blood cultures, testing and reporting of susceptibility.

**Session 2** (Penicillins & Pneumonia)

- Penicillins: Mechanisms of action, differences between penicillins, side effects, dosing, PK/PD.
- Pneumonia: Clinical manifestation, risk stratification, microbiological diagnostic testing, expected pathogens, empirical and focused therapy.

**Session 3** (Cephalosporins & Perioperative prophylaxis)

- Cephalosporins: Mechanisms of action, differences between cephalosporins, side effects, dosing, PK/PD.
- Perioperative prophylaxis: indication for prophylaxis, antibiotic choice, timing of administration, duration of prophylaxis.

**Session 4** (Vancomycin & Bloodstream infections)

- Vancomycin: Mechanisms of action, side effects, dosing with emphasis on TDM, PK/PD.
- Bloodstream infections: Clinical manifestation, risk stratification, microbiological diagnostic testing, importance of counselling, therapeutic principles

**Session 5** (Carbapenems & Sepsis)

- Carbapenems: Mechanisms of action, differences between carbapenems, side effects, dosing, PK/PD.
- Sepsis: Definition, microbiological diagnostic testing including timing of specimen collection, expected pathogens, risk for multi-resistant pathogens, empirical and focused therapy.

**Session 6** (Macrolides & Skin and soft tissue infections)

- Macrolides: Mechanisms of action, differences between macrolides, side effects, dosing, PK/PD.
- Skin and soft tissue infections: International differences in nomenclature, clinical spectrum of disease, principles of diagnosis, non-infectious differential diagnoses, empirical and focused therapy.

**Session 7** (Chinolones & Urinary tract infections)

- Chinolones: Mechanisms of action, differences between chinolones, side effects, dosing, PK/PD.
- Urinary tract infections: Clinical manifestation, risk stratification, microbiological diagnostic testing, treatment principles including when not to treat.

**Session 8** (Penicillin allergy & case-based wrap-up)

- Penicillin allergy: clinical presentation, stratification according to severity, epidemiology, delabeling.
- case-based wrap-up: diagnosis and therapy of selected exemplary cases, penicillin allergy delabeling decisions of exemplary cases.
